# Can Social Prescribing Foster Individual and Community Well-Being? A Systematic Review of the Evidence

**DOI:** 10.3390/ijerph18105276

**Published:** 2021-05-15

**Authors:** Dragana Vidovic, Gina Yannitell Reinhardt, Clare Hammerton

**Affiliations:** Department of Government, Faculty of Social Sciences, University of Essex, Colchester CO4 3SQ, UK; gina.reinhardt@essex.ac.uk (G.Y.R.); chamme@essex.ac.uk (C.H.)

**Keywords:** social prescribing, public health, isolation, loneliness, well-being, connectedness, systematic review

## Abstract

Social prescribing programmes (SP) are person-centred coaching schemes meant to help participants improve individual circumstances, thereby to reduce demand on health and social care. SP could be an innovative means to improve preventive and public health in the pursuit of universal financially sustainable healthcare. Given its potential, our systematic review assesses type, content, and quality of evidence available regarding SP effectiveness at the individual, system, and community levels. We examine the impact of SP on addressing loneliness, social isolation, well-being, and connectedness, as well as related concepts, which are not yet considered jointly in one study. Following PRISMA, we search: EBSCOHost (CINAHL Complete; eBook Collection; E-Journals; MEDLINE Full Text; Open Dissertations; PsycARTICLES; PsycINFO); Web of Science Core Collection; and UK National Institute for Health and Care Excellence. Excluding systematic reviews and articles without impact evaluations, we review 51 studies. Several studies do not distinguish between core concepts and/or provide information on the measures used to assess outcomes; exactly one peer-reviewed study presents a randomised controlled trial. If we wish to know the potential of social prescribing to lead to universal financially sustainable healthcare, we urge researchers and practitioners to standardise definitions and metrics, and to explore conceptual linkages between social prescribing and system/community outcomes.

## 1. Introduction

The United Nations Sustainable Development Goals (UN SDGs) include a focus on “ensuring healthy lives and promoting well-being for all at all ages” [1]. As part of the pursuit of universal health coverage and sustainable health financing, some countries have begun to focus on well-being as a target. Because well-being is closely associated with physical, mental, and public health outcomes, efforts to support well-being are seen as potential avenues for preventing physical health problems, lowering the risk of public health emergencies, reducing the overall cost of healthcare, and promoting public health.

Practitioners, policy makers, and researchers around the world have been calling for an innovative and fundamental change in healthcare systems as a means to encourage well-being, reduce loneliness and isolation, and improve connectedness among individuals. One increasingly popular approach to addressing loneliness and social isolation, and to improving individual health and well-being and community connectedness, is social prescribing [2]. Social prescribing is a community-based, person-centred, holistic health coaching scheme, which supports individuals to better understand their needs and take action to improve their health and well-being [3,4]. It empowers individuals to identify their own needs and find solutions in a way that is meant to give individuals choice and control over their mental and physical health, improve public health and well-being, and enhance value for money [5]. The growing interest in social prescribing has led to calls for researchers and programme managers to evidence the impact and articulate the ways in which social prescribing models are expected to work [6].

To answer this call for evidence and the call for research into innovative preventive services that promote health, we conduct and present a systematic review of evaluations of social prescribing programmes that are designed to cause reductions in loneliness and social isolation and improvements in well-being and connectedness. We focus on the quality and content of studies that address mechanisms and outcomes of social prescribing initiatives to provide insight into the extent to which person-centred care delivery models can address new challenges in promoting health with innovative approaches to preventive healthcare services. 

### 1.1. Key Outcomes 

Loneliness, a subjective, unwelcome feeling of lack or loss of companionship, occurs when there is a mismatch between the quantity and quality of social relationships that a person has, and those that a person wants [7,8]. While loneliness is likely to be experienced by most people at some point in their lifetime, it is the chronic condition of loneliness that has garnered attention in social sciences, and in various government, community, and voluntary sector organisations. This interest is driven by reports that link persistent feelings of loneliness to a variety of physical and mental health issues and early mortality risks [8,9]. It is believed that loneliness is contributing to an increase in usage of public health and social care services [10]. Given the potential loneliness-related impacts, the United Kingdom (UK) government has recognised loneliness as a threat to public health and is addressing the issue via a Loneliness Strategy, appointing a Minister of Loneliness, and numerous resources to combat loneliness [11,12]. Around the globe, governments are seeking solutions to address loneliness and social isolation [13].

Social isolation, an objective, quantifiable measure of the number and the quality of contacts that one has, is another global issue that influences health and well-being [14]. While related to loneliness, social isolation is a distinct phenomenon; socially isolated individuals do not necessarily experience loneliness, and lonely individuals do not necessarily have less social contact with others [15]. Both phenomena are linked to depression, a mental health condition that impacts physical health, well-being, and ways in which one adjusts and recovers from difficult life experiences [15]. As such, these phenomena present an opportunity for innovative health solutions to address. Initiatives that aim to improve satisfaction with one’s social network and the support that the network offers may be able to motivate people to engage in meaningful and productive activities with others, give them a sense of purpose, and help improve overall well-being. 

Well-being is a personal or subjective feeling about one’s life, and is a combination of a personal sense of satisfaction with life, a sense that what we do in life is worthwhile, and how happy and anxious we are [16]. These four aspects of well-being are commonly included in national and international surveys. The concept and measures of well-being can be expanded to include functional aspects (feelings of autonomy), vitality (sleeping and eating habits), and feelings about the community in which one lives [17]. The complex nature of well-being presents challenges for studies that seek to link well-being to various dimensions of health [16] because the components of health and well-being are often co-determined [18] and related to one’s active participation in social, economic, and political life [19]. Given the complexities of this relationship, governments are increasingly recognising the need to address health *and* well-being as essential for preventing deteriorations in physical, mental, and public health [19,20]. 

Connectedness is another outcome of interest with respect to public and preventive health. In their 2018 report, New Zealand’s Ministry of Social Development named connectedness as a key driver of well-being and resilience [21]. While there is no generally accepted definition of connectedness, it can be described as an experience of belonging and relating to others [21,22]. Many preventive health programmes and social prescribing initiatives focus on improving connectedness, though a lack of conceptual clarity, which we present below, presents challenges to assessing levels of connectedness and potential changes therein. Measures of connectedness range from counting an individual’s group activity attendance and perceived group support [23] to assessing an individual’s sense of acceptance [24], belonging, or identity [25], and to consideration of connectedness in terms of its relation to loneliness and social isolation [26]. In addition to these conceptual challenges, we also lack a theoretical explanation as to precisely why and how social connectedness enhances health [27]. 

### 1.2. Effects at Multiple Levels

Together, these four concepts represent a potential target for preventive health initiatives on multiple levels. Individuals experiencing higher levels of well-being are both less likely to become ill and more likely to be able to continue their own economic activity and help others during a public health emergency [28]. This means higher levels of well-being should be associated with the ability to weather pandemics, such as that driven by COVID-19, and to bounce back from the shocks associated with such a pandemic. People who are socially connected to their communities, and communities of people with high levels of social connection, should be better at communicating quickly and more able to work together during times of stress and uncertainty [29]. People who have the low levels of trust in others that often accompany loneliness will have a harder time accepting the advice or help of others, even people tasked with managing crises by providing guidance [30]. Initiatives that address these issues, then, should help prevent worse health outcomes and public health crises later. 

Reducing loneliness and isolation and increasing trust and connectedness are positive individual-level changes that programmes such as social prescribing are meant to bring about for participants. Such programmes are also, however, meant to spark positive changes at the system and community levels, meaning evaluations of programme effectiveness should include these levels as well [3,31,32]. The argument is that improvements in individual-level health and well-being will help individuals gain confidence, retain memory, and even remain autonomous longer, meaning they are less likely to need long-term health or residential care [10,33]. As people feel better about themselves and their lives, they are also less likely to call on medical services for non-physical needs, such as loneliness and depression. Medical and social services should experience more targeted and, ultimately, reduced demand. 

Further, people who are more connected to their communities and more trusting of others should contribute to overall community connectedness, thus enhancing a community’s social capital. With higher levels of connectedness, people should be more likely to help each other in times of need, to establish networks that trade favours, and to generally be healthier for longer [33]. Communities that are more connected are therefore argued to be more economically productive, to be less likely to need social and public services, and to have lower crime rates [33]. At the individual, system, and community levels, interventions like social prescribing are meant to prevent crises and promote health.

### 1.3. The Need for Evidence

Although connections between these types of programmes and preventive health are plausible, we know very little about the impact of initiatives such as social prescribing that are seeking to address them. Social prescribing has been present in the UK since the 1970s [24] and is a key means with which the UK Government aims to support 2.5 million people by 2023/24 to manage their own health, improve their well-being, and reduce both pressure and spending on health and social care services [34]. Some estimate that 20% of the general practice (GP) appointments in the UK are related to social issues [35], while this estimate is closer to 30% in the Netherlands [36]. Given the existing evidence and the budgetary constraints that many services face, social prescribing initiatives are beginning to be implemented in other countries, including Australia, Canada, Denmark, the Netherlands, and Norway [25,36,37,38,39,40,41], as potential avenues for improving public health and preventing health crises.

Despite its popularity, reports indicate that scientifically rigorous and validated evidence of the impact of social prescribing is sparse [3,6]. Few studies implement internally or externally valid research designs to connect social prescribing programmes to changes in loneliness, isolation, well-being, connectedness, demands on health and social care, community-level changes, or other outcomes associated with public and preventive health. Notwithstanding the lack of systematic evidence of social prescribing’s impacts on individuals, systems, or communities, there is enthusiasm for these types of programmes from both service delivery personnel and programme participants [42,43,44]. To glean insights and guidance, programme administrators are forced to turn to reports and studies that are not peer-reviewed and do not include randomised controlled trials (RCTs) or other recognised means of causal inference. 

The potential and interest in social prescribing is driven by the person-centred approach, support planning that is based on one’s needs, resources, and strengths, and the methods to empower individuals to actively participate in decisions regarding their health and well-being. This model of personalised care is delivered by social prescribing professionals who are experienced, informed, and knowledgeable about community resources. They are referred to as link workers (community connectors), who are trained to assist individuals to navigate their health needs and resources available to address them [5]. The key aspect of any social prescribing programme is this interaction between the link worker and the individual (or a carer) over the course of one’s programme participation. This interaction distinguishes social prescribing from other community-based approaches to health promotion and disease prevention [45]. Most importantly, it is the quality of this relationship that is argued to be the reason for choosing social prescribing as the person-centred approach to use and is cited as a key reason for some of the social prescribing programme successes [43,44]. Still, this lack of evidence raises questions regarding the ethics and feasibility of delivering social prescribing as a strategy to improve public and preventive health. Some health care providers have expressed reservations about referring patients to a service that might not have the same level of quality control or expertise regarding confidentiality and safeguarding as national health services would [42]. 

Based on the need for innovative preventive public health services to address issues such as loneliness, isolation, well-being, and connectedness and the marked increase in social prescribing as a proposed solution to this need and subsequent demand for rigorous, externally validated research on the topic, we present this systematic review. Our work contributes to the Special Issue on New Challenges and Crucial Topics for 2030 Public Health by providing insight into social prescribing initiatives and their potential to promote public and preventive health by affecting loneliness, isolation, well-being, and connectedness, and by affecting individual participants, health and social care system-level demands, and community-level outcomes. We systematically review the literature on social prescribing over the last 20 years and synthesise the results by identifying: (1) the extent of evidence of social prescribing impact on individual participant loneliness, social isolation, well-being, and connectedness; (2) the type and quality of evidence used to demonstrate impact; and (3) the impact of social prescribing programmes that are designed to improve our four key outcome measures on public and preventive health challenges at the individual, system, and community levels. 

Our efforts build on previous work in at least two important ways. First, our consideration of loneliness, isolation, well-being, and connectedness as four distinct items of interest is a focus we have not seen in previous work, which systematically reviews the effects of social prescribing on physical health, healthcare usage, and well-being [45,46], or which examines only loneliness as an outcome [47] but does not address isolation or connectedness. Second, we report on these outcomes of interest at the individual, system, and community levels. We find that a lack of evidence regarding how social prescribing works, and for whom, is partially due to how programme impact is assessed and categorised and partially due to how evaluations are designed and conducted. Our work thereby expands the conversation and restricts the claims about the impact and effectiveness of programmes such as social prescribing.

## 2. Materials and Methods

We conduct our review following the guidelines set in Pettigrew and Roberts [48] and report findings following the Preferred Reporting Items for Systematic Reviews and Meta-Analyses (PRISMA) guidelines [49]. The protocol is available from the authors on request. 

### 2.1. Research Strategy

Our comprehensive search combed social science and public health repositories to identify studies on the effect of social prescribing on loneliness, social isolation, connectedness, and well-being. We searched publications with dates ranging from 1 January 2000 to 6 October 2020, identifying research articles and reports published in the English language. While the idea of social prescribing emerged before 2000 in the UK, the attention on social prescribing as one of the models to be used to promote good health, as it exists today, is becoming more evident following a 2006 UK government white paper report [50]. Moreover, the global interest in similar types of initiatives is only becoming apparent with the World Health 2000 report calling for the “intersectoral initiatives” to improve people’s understanding of health, health service provision and delivery, and the awareness of individuals as active participants and co-producers of their health [51]. We therefore begin our search with publications from 2000, which would have followed the World Health Organisation Reports on 21st Century health priorities [51,52,53], and which will make our work easily comparable to previous systematic reviews that also begin at 2000 [45,46]. 

We searched nine databases. Seven of these were accessed using EBSCOHost: CINAHL Complete; eBook Collection; E-Journals; MEDLINE with Full Text; Open Dissertations; PsycARTICLES; and PsycINFO. The two additional databases are the Web of Science Core Collection and the UK National Institute for Health and Care Excellence (NICE). EBSCOHost and Web of Science Core Collection include peer-reviewed scholarly journals published worldwide (including open access journals), as well as conference proceedings and books. NICE includes reports issued by think tanks, non-profit organisations, community health groups, and the government, as well as social science and medical journals such as *The BMJ* that have national and international reach [54]. The databases were selected on the basis of their usage in social sciences literature (Web of Sciences, EBSCOHost) as well as social prescribing literature (NICE, CINAHL, and MEDLINE via EBSCOHost). To these, we also added a search for grey literature on the Future NHS collaborative platform to capture reports published internally by research centres and shared online [54]. At Future NHS, practitioners and researchers post-peer-reviewed and non-peer-reviewed reports on social prescribing and its evaluation. Practitioners and administrators, in the absence of peer-reviewed work, often turn to grey literature for guidance. We therefore include these sources in our review and assess the quality of evidence therein. 

**Figure 1:**
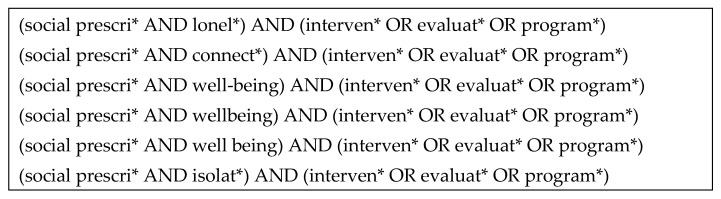
Search strategy used in the systematic review of social prescribing programmes on loneliness, social isolation, connectedness, and well-being. * indicates word root in search, meaning any suffix would be captured.

Our search terms included a combination of loneliness, social isolation, connectedness, well-being, social prescribing, evaluation, intervention, and programme. We arrived at this list after examining alternative search strings, including terms such as project, result, pilot, and impact, and selected the search string that produced the largest number of results (Figure 1). Each of the 6 search strings (statements) presented in Figure 1 was entered individually to each of the 9 databases, as well as into the search function of the Future NHS platform. 

**Figure 2:**
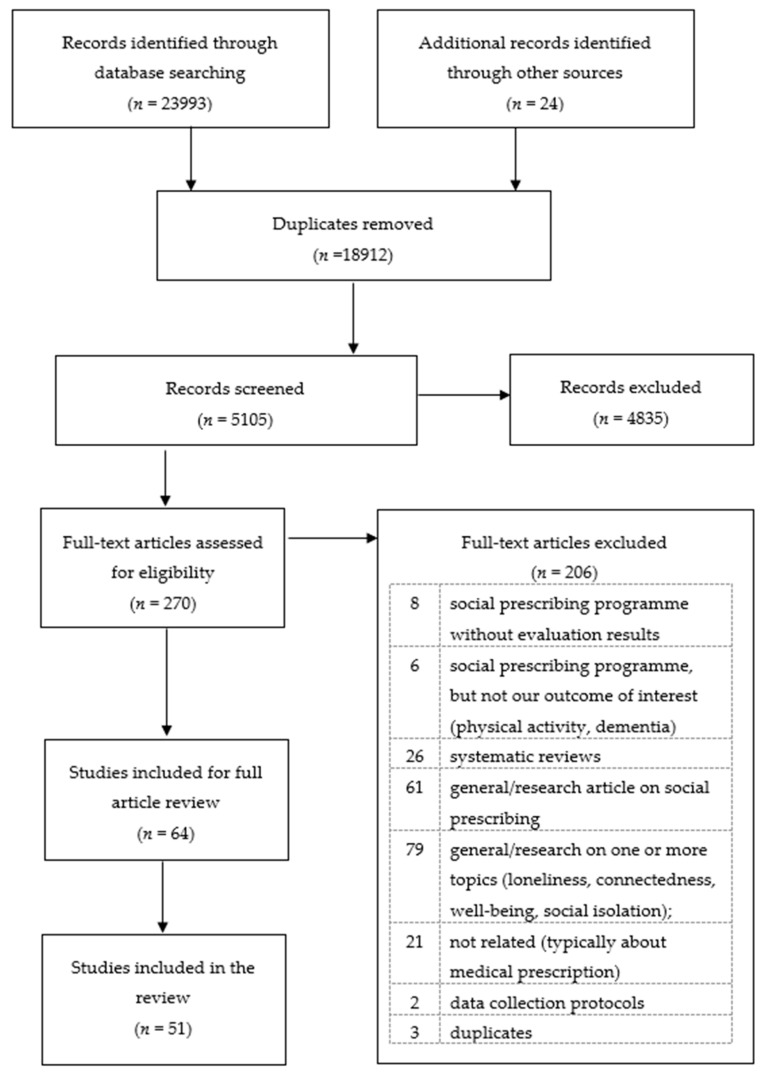
PRISMA flow diagram of the systematic review of social prescribing programmes designed to address loneliness, social isolation, well-being, and connectedness across the globe, 2000–2020.

We restrict our search to terms related to ‘social prescribing’ rather than other forms of community-related or person-centred health interventions because of the recent surge in programmes specifically designated to be social prescribing. While the search was not limited geographically, it is possible that the ‘social prescribing’ term has been more commonly used in the UK in the past; however, this trend has changed in recent years, with the international community being more aware of this model of community intervention [13]. In addition, Figure 2 shows that our search identified a number of articles that are not linked to social prescribing but are related to other research and community aspects of loneliness, social isolation, well-being, and connectedness. 

### 2.2. Data Synthesis 

The authors independently assessed the full text of each of the 51 studies and extracted details into a database. Data collected for each study were: authors; social prescribing programme name; location; evaluator; research design; method; impact measures; and a detailed summary of the findings regarding impact on the person, the health and social care system, and/or the community. Table S1 provides this comprehensive information. The outcomes of interest for our review were loneliness, social isolation, well-being, and connectedness. 

### 2.3. Inclusion Criteria and Study Identification

Figure 2 shows our search according to PRISMA diagrammatic rules. Our initial search obtained 24,017 (23,993 + 24) articles, of which 280 were identified via EBSCOHost, 636 via Web of Science Core Collection, and 23,077 via NICE, for a total of 23,993 records. Our search of grey literature resulted in 24 articles and reports. We identified and removed 18,913 duplicates using the Zotero ‘duplicate items’ option, resulting in 5104 articles remaining for screening. In the first step of screening, the abstracts of these 5104 distinct articles were reviewed by the authors to identify studies including a programme or initiative designed to offer person-centred care. Out of these 5104, 4834 articles were excluded due to being reports on clinical outcomes (such as cancer or heart disease), general health, or social care. In total, 270 articles proceeded to the second stage, which involved full-text assessment to identify studies evaluating the impact of one or more social prescribing intervention on loneliness, social isolation, well-being, and/or connectedness. 

We include non-peer-reviewed studies in this systematic review, as a number of these studies are used to inform national and local health policies. Social prescribing as a field of study is underdeveloped in both theoretical and methodological aspects, with the current ‘link worker’ model becoming more widely used and studied only in recent years. Thus, the literature on social prescribing should be considered to be at its inception. A systematic review of this literature is thus timely and important. Given our timeliness, mapping and summarising both peer- and non-peer-reviewed studies illustrate the efforts to build an evidence base in this field. Though many of the non-peer-reviewed studies follow the most recent national guidelines and recommendations in regard to their evaluations, we include them not because we assume they are of high quality, but because we know that their results are informing national and local health policies. As an example, Evaluation of Doncaster Social Prescribing Service [55] is referred to in the UK Government Loneliness Strategy policy paper [11]. We therefore show and evaluate the type of evidence that is available for those looking to design and implement a social prescribing programme. The field of social prescribing is currently grappling with a need to build a robust and informative evidence base on the impact of social prescribing on outcomes of interest [6,45,56]. Our goal is to provide a complete and detailed synthesis of the evidence as it currently exists in the field and an evaluation of the extent to which this evidence should be used to inform the process of building a social prescribing evidence base.

At this stage, 206 studies were excluded because they: did not include evaluation results (8); did not evaluate one of our four outcomes of interest (6); were systematic reviews (26); were general articles on the topic of social prescribing (61); were general articles on one or more of our outcomes of interest (79); were not about social prescribing (21); were data protocols (2); or were duplicates (3). This process left 64 studies remaining for full article review, at which point the authors excluded 13 that were: study protocols with no evidence (2); a scoping review (1); a general article on one of our topics of interest that did not include an intervention (1); a systematic review (1); general articles on social prescribing (2); evaluations of a service that was not a social prescribing programme (2); and not related to our outcomes of interest (4). When coding disagreement occurred, the article in question was discussed by multiple members of the team to reach a final decision. With the remaining 51 articles that include social prescribing intervention evaluation results, we identified the impact of the intervention under study on one or more levels of analysis: the person, the health and care system, or the community. 

## 3. Results

### 3.1. Study Characteristics

The 51 studies are based on social prescribing initiatives conducted from 2014 to 2020. Of these, 33 are published in peer-reviewed outlets and the remainder are study reports. In addition to the UK, our search identified initiatives implemented in Australia, Denmark, the Netherlands, and Norway [25,36,37,38,39,40]. Within the UK, social prescribing programmes were implemented primarily in England, with three in Scotland [42,44,57] and one in Wales [47]. Those in England were widely spread across the region. Please see Supplementary Table S1 for full details of all studies. 

The social prescribing projects studied include a total of 41,641 participants (aged 18 or older). Thirty-six studies employ a pre/post design [26,27,37,38,39,44,55,57,58,59,60,61,62,63,64,65,66,67,68,69,70,71,72,73,74,75,76,77,78,79,80,81,82,83,84,85], 8 report case studies with evidence taken at one point in time [26,42,70,74,78,83,85,86], 16 studies conduct surveys [27,37,55,57,58,60,62,63,68,72,77,78,80,83,84,87], 14 conduct interviews [24,25,36,39,40,43,44,74,85,88,89,90,91,92], 17 mix both survey and interview methods [26,38,42,59,61,66,67,69,70,71,73,75,76,79,81,82,92], 7 conduct thematic analysis [43,44,71,88,90,92,93], and 5 employ focus groups [25,38,62,87,92]. Three studies employ an experimental or quasi-experimental design that includes some form of control group comparison or randomization [57,60,81]. 

### 3.2. Outcome Measures

**Figure 3:**
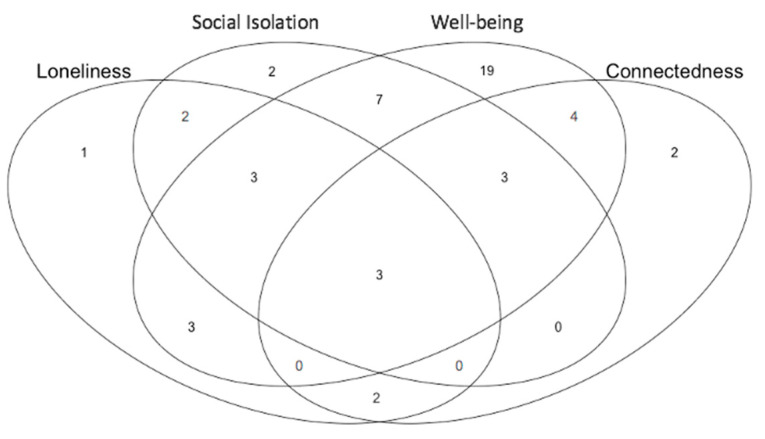
Venn diagram of the 51 papers according to which outcomes they address of the 4 key concepts that are the focus of this systematic review: loneliness, social isolation, well-being, and connectedness.

We are principally concerned with studies that examine the impact of social prescribing on four key concepts: loneliness, social isolation, well-being, and connectedness. Before summarising the articles in terms of measurement and evidence quality, we offer a Venn diagram of the 51 studies according to which of the 4 outcomes they measure (Figure 3). Fourteen of the fifty-one studies assess loneliness as an outcome of interest [26,27,37,58,74,75,76,78,79,80,85,87,89,92]. Of these, seven use a version of the UCLA (University of California, Los Angeles, CA, USA) scale to measure loneliness [26,27,37,79,80,85,89], one uses the Delphi method to conduct thematic analysis [87], and one uses the Hawthorne Friendship Scale [76]. Four either do not report how they assess loneliness or do not report how their assessments were designed or chosen [58,74,75,92]. 

Social isolation appears to be the least developed outcome, both conceptually and operationally. The impact of social prescribing on social isolation is therefore particularly under examined, although it is one of the more common reasons for referral to a social prescribing programme (see Supplementary Table S1). Of the 20 studies with a stated interest in assessing social isolation, 2 use interview quotations and analysis to gauge impact [66,82], 2 conduct thematic analyses [88,92], and 4 conflate the concept with loneliness [26,66,78] or connectedness [64] and believe they measure it using the same indicator/metrics. Nine either do not report how they assess social isolation or do not report how their assessments were designed or chosen [36,42,43,58,59,61,70,74,75,94].

Forty-two of the studies are concerned with well-being. While 14 studies rely on either the long [26,38,42,59,63,66,67,70,71,73,77,81] or short [80,83] version of the Warwick–Edinburgh Mental Well-being Scale (WEMWBS), others employ measures such as the UK Office for National Statistics 4-question (ONS4) measure [68,76]; the University College London (UCL) Museum well-being measure [82,85]; interview and survey questions designed for the purposes of a particular programme [64,65,69,75,78]; the Kessler Psychological Distress Scale (K10) and the World Health Organisation Quality of Life score (QoL) [37]; self-determination theory [44]; psychological measures of depression and anxiety [60]; the Medical outcome profile (MYMOP) [61]; the Investigating Choice Experiments for the Preferences of Older People Capability Measure for Adults (ICECAP-A) capability-based measure [57]; or tests for components of well-being, such as self-confidence, strength, resilience, joy, and vitality [27,28,33,37,72]. Nine studies do not provide details of how well-being is assessed [36,47,53,57,62,68,70,82,87].

Fourteen studies consider connectedness [24,25,26,27,55,66,75,76,79,82,83,84,90,93]. Of these, three conflate the concept with loneliness [26,55,66] and four either do not report how they assess connectedness or do not report how their assessments were designed or chosen [75,76,84,90]. Other studies use measures such as the UK Adult Social Care and Public Health Outcome Framework [55], the UK Campaign to End Loneliness measure of social networks [66], surveys [27,79], interview quotations [82], or survey/interview questions developed for the purposes of a particular programme [24,25,83,93].

Table 1, Table 2 and Table 3 summarise the articles according to measurement data and evidence quality. We find that the 4 outcomes are addressed with varying success in the 51 studies. Of the 27 studies aiming to address 2 or more of these outcomes, 4 either do not distinguish between loneliness, connectedness, and isolation or use the terms interchangeably [26,55,66,78]. This finding illustrates an ongoing challenge with defining and measuring the outcomes of interest and, hence, with determining the overall impact of social prescribing.

### 3.3. Impact on the Individual

All 51 studies assess the impact of social prescribing on the individual. Table 1 summarises the information offered regarding individual-level impact, providing a list of measures used to assess impact and the number of studies using each measure. The third column of Table 1 identifies the studies that provide evidence of change in loneliness [27,37,79,80,85], social isolation [60,66,76], well-being [26,37,38,44,55,57,59,60,61,63,64,65,66,67,68,69,70,71,73,75,76,77,78,80,81,82,83,85], and connectedness [27,79,82]. This column contains studies that provide clearly defined, verifiable, standardised measures of the concepts and outcomes of interest and an assessment of change such as pre- and post-programme comparisons. Other studies either lack pre–post comparison design or conceptual clarity or do not include enough information regarding measurement to enable an assessment of evidence quality. One study assesses programme impact by gathering feedback from the social prescribing delivery staff, who have identified issues such as programme length and a better understanding of the scope of social prescribing as key to successfully addressing a particular outcome of interest [80]. 

The fourth and final column includes studies that make causal inferences based on either an internally valid research design (RCT) or a quasi-experiment that has accounted for potential alternate variables or confounding factors by using statistically sound analytical methodologies. We classify the findings into these two categories to distinguish between change and causality. Whereas the studies in the third column show either statistically significant or qualitatively verifiable evidence of change in a key outcome measure, the studies in the fourth column are able to link the change that they find to participation in a particular social prescribing programme.

Table 1 findings show the variability in the types of measures used in regard to the four outcomes, with half the studies on loneliness and well-being utilising comparable measures and measures of social isolation and connectedness being even more varied. We also see that only a small number of studies provide evidence of change, with only 5 out of 14 loneliness studies in this category, 3 out of 20 for social isolation, 28 out of 42 for well-being, and 3 out of 14 for connectedness. There are even fewer studies that offer means of causal inference, with only five studies across all four outcomes of interest in this category. Two out of these five studies are based on the Social Cure programme [27,79]. One of the Social Cure studies, Kellezi et al. 2019 [79], is the first study to our knowledge that shows a link between various concepts of individual-, system-, and community-level effects. The authors show that an increase in the number of group memberships between t_0_ (M = 1.89, SD = 1.59) and t_1_ (M = 2.21, SD = 1.87, F (1177) = 5.34, *p* = 0.022, partial η^2^ = 0.029) is a positive predictor of community belonging at a follow-up (t_1_ = 0.09, *SE* = 0.04, *t* = 2.61, *p* = 0.01, *LLCI* = 0.02, *ULCI* = 0.16), while community belonging at the follow-up is a negative predictor of loneliness at follow-up (t_1_ = −0.31, *SE* = 0.07, *t* = −4.15, *p* = 0.0001, *LLCI* = −0.45, *ULCI* = −0.16), which is a positive predictor of healthcare usage at follow-up (t_1_ = 1.41, *SE* = 0.45, *t* = 3.13, *p* = 0.002, *LLCI* = 0.52, *ULCI* = 2.31). These findings indicate that individuals that are members of more groups are likely to have a greater sense of community belonging, lower loneliness levels, and are likely to use health care services less (see more in Table S1). 

Most of the other studies also find statistically significant change in outcomes at the individual level; however, given the types of statistical comparisons and a wide range of reported changes, it is not possible to draw clear conclusions of the impact. The change as reported in percentages ranges from 46–69% of participants feeling less lonely [78,80] and 19% feeling less socially isolated to 71–94.7% reporting improvements in well-being [58,62,66,80,84] and 50.7–95% reporting a greater sense of connectedness (see Table S1 for more details) [66,84]. Three studies find no statistically significant change in outcomes at the individual level. Evaluators suggest that better post-programme data [59], theoretical/empirical improvements [37,57], and more appropriate comparison groups would be useful in further research.

The utility of the evidence differs across studies as well. Out of the 10 studies that describe their loneliness measure, 7 employ a version of the UCLA loneliness scale. Similarly, 14 of the 33 studies that describe their well-being measure employ the WEMWBS scale. This consistency in measure selection lends itself to cross-study comparisons that could help improve our understanding of social prescribing effects. Additionally, studies that employ less commonly used measures and methods offer useful insights into the complex nature of the issues. In the case of well-being, a study utilising the Investigating Choice Experiments for the Preferences of Older People Capability Measure for Adults (ICECAP-A) and a quasi-experimental cluster-randomised controlled trial finds no improvements in well-being [57], while another study applies the self-determination theory to assess change and finds improvements in well-being for the same programme [44]. 

Though evidence of individual-level change in the outcomes of interest is generally robust, evidence that a social prescribing programme caused the change in outcomes is rare. In the fourth column of Table 1, we identify only five studies that can claim a causal link between a social prescribing intervention and changes in key outcomes through robust means such as control group comparisons, randomisation, or statistical methods for causal inference such as mediation analysis or propensity score matching [27,57,60,79,81]. Due to the lack of robust causal evidence, we cannot conduct a meta-analysis of the effects of social prescribing, and we are limited in the conclusions we can draw regarding the impact of social prescribing on individual outcomes. 

### 3.4. Impact on the System 

Impact on the system is the second most common level of impact identified, with studies on health care usage accounting for the majority of the 22 studies in this category (see Table 2). Most of the studies examine self-reported health care [26,55,60,61,66,67,76,78,79,83], accident and emergency (A&E) visits [55,58,67,68,73,74,75,76,78,83,95], and hospital admissions [55,68,73,75,76,78,95]. Following social prescribing programme implementation, eight studies report a reduction in health care appointments [54,59,60,69,70,72,73,77], nine report reduced A&E attendance [51,60,61,66,67,68,69,70,72,77], and seven find reduced hospital admissions [55,68,73,75,76,78,95]. Two of these twenty-two studies examine mental health service usage, with one study finding an increase in mental health service discharge rates [64] and another finding reduction in mental health service usage [55]. Other studies focus report changes in the number of outpatient appointments [74,75,76], cost savings [55,58,60,63,73], and effectiveness of referral pathways and hospital discharge rates [72].

Just as with individual-level outcomes, the degree of change differs across studies. Reported reductions range from 7–68% reductions in GP usage [55,83] to 7–50% reductions in A&E usage [55,68]. Interestingly, one study has found that GP service usage stayed the same for roughly half of the participants (53.3%) while it decreased for the rest [66]. Only two studies find no statistically significant impact on GP usage [26,53], and one study finds an increase in A&E usage [95]. Overall, the primary interest in impact on the system is in relation to health service usage and the associated cost-benefit analyses. Three studies investigate the impact of a social prescribing programme on social care [55,63,92].

Studies that consider the system level are also rarely able to establish a causal link between social prescribing and the outcomes they find. One of the studies that examines social care usage is able to causally link social prescribing to a reduction in costs of social care [63]. One study utilising randomised controlled trials finds no statistically significant impact of social prescribing on health care usage [60]. Another study employs mediation analysis to conclusively link participation in a social prescribing programme to a reduction in loneliness levels and in turn to a reduction in healthcare usage [79]. Three other studies offer insight into the pathways through which impact can be achieved, the challenges social prescribing programmes are likely to face, and the views of health professionals on the value of social prescribing in reducing the demand on health care services [42,69,79].

### 3.5. Impact on the Community 

Eighteen of the fifty-one studies consider impact at the community level [26,36,42,44,55,58,67,69,73,74,75,76,79,80,82,89,92,95]. Table 3 illuminates the need for conceptual and methodological progress in this area, revealing the lack of clarity and conceptual linkage between the measures employed and the key outcomes of interest. We find 15 studies that could be categorised as considering community resources [26,36,42,44,55,58,67,69,73,74,75,76,79,80,82,89,92,95] and eight of which provide evidence of change, with none successfully linking the change in outcomes to a particular social prescribing intervention in regard to the community resources concept. Three studies consider community connectedness as an outcome of interest [69,79,95], with three providing evidence of change [27,69,79], and two of these studies which are based on the Social Cure programme being able to establish causal links in regard to the community connectedness/belonging concept. The question used to assess community belonging in Social Cure studies, ‘Thinking about this local community, the kind of place it is and the kind of people who live around here, would you say that you feel a sense of belonging to this local community?’, defines community connectedness as a sense of belonging (See Table S1 for more details). 

Given the lack of conceptual and methodological clarity, future studies should focus on building and expanding theoretical frameworks and developing measures that could be used to capture the impact of a social prescribing intervention on a community. A number of studies provide guidance in this regard (see Table S1) [26,69,73,74,76,79,89,92]. For example, a study on the Community Navigation programme explains the potential impacts of a social prescribing programme on community aspects, such as local mental health and well-being strategies, health access, community cohesion, social capital, volunteering opportunities, and skills development [69]. 

## 4. Discussion

Overall, a majority of studies in this report find change *following* a social prescribing intervention, but not necessarily change *due to* a social prescribing intervention. At the individual level, we find that evidence of change is clearer in regard to loneliness and well-being in comparison to social isolation and connectedness. At the system level, we find that evidence of change predominantly pertains to health care, with very little attention to social care. At the community level, change is most evident in terms of community resources, with some work also addressing connectedness. The reported increased changes illustrate the potential for improvements in public health. Yet we find that only five studies at the individual level [27,57,60,79,81], two studies at the system level [60,79], and two studies at the community level [27,79] provide evidence to support claims that the observed change is due to a particular social prescribing programme. Given this small number of studies that establish causality, conclusions regarding the impact of social prescribing are tentative, at best.

Our findings point to at least two opportunities to improve our understanding of the impact of social prescribing. We recommend careful consideration of current and future social prescribing research practice in order to capitalise on these opportunities. We do identify a number of studies upon which future studies and social prescribing intervention can model their approaches. The studies able to successfully claim that changes in outcomes are due to a social prescribing intervention are those that use a research design that allows for the above-recommended pre- and post-comparisons between treated and untreated groups or those that use statistical methods of causal inference, such as propensity score matching or mediation analysis, to arrive at their findings. Though currently small in number, these studies offer valuable lessons regarding the potential impact of social prescribing programmes.

Now that social prescribing has become part of the national strategy of the UK and is being considered elsewhere as a means to improve overall preventive and public health, improving our evidence base is critical to informing preventive public health decisions moving forward. Based on the examples offered by the rigorous studies we found, we divide our recommendations into two general areas: programme design and programme delivery. Ultimately, we recommend standardising measurement and data collection to help deliver stronger, more reliable, and more rigorous evidence.

### 4.1. Programme and Design and Measurement Selection

One of the biggest areas for improvement in programme design thus lies in conceptual development. Given the potential of social prescribing to affect so many areas of individual and community life, it can be easy for proponents and advocates to think about an enormous array of potential programme outcomes and impacts. With such a wealth of potential goals, any one programme could be targeted toward any number of outcomes. The resultant diversity in which outcomes should be assessed then leads to confusion in terms of the conceptual development of some outcomes. Loneliness, well-being, and health care service usage have high levels of conceptual and methodological consensus and clarity; people know what the concepts mean, and they agree on ways in which the concepts can or should be measured. In contrast, concepts such as social isolation, individual connectedness, and community connectedness are often conflated with each other or with loneliness, well-being, and social capital. As a result, sometimes even those delivering the project do not know how they would evidence a change in the outcomes of interest.

A full exploration of how individual changes in well-being lead to changes in health system usage, or in community-level productivity, are even more difficult to find. Despite evidence of change in health care usage, a lack of discussion on the processes through which this change is achieved precludes a greater understanding of potential programme impact, as well as its value as an innovative approach to achieving financially sustainable universal health. Similarly, although our review identifies a variety of community-related outcomes that revolve around connecting individuals with each other and their communities, building community cohesion and social capital, there is no agreement on the impacts or community assets that are specifically related to this outcome.

We therefore recommend that social prescribing programme designers carefully document, in the design phase, exactly which outcomes are targeted, how those outcomes will be measured, and how the outcomes as concepts are theoretically linked to the measures chosen. Studies of the Social Cure social prescribing programme offer an excellent example of this specification. The authors utilise well-justified and validated measures to assess and test the links that exist between an individual’s sense of loneliness and belonging and its impact on healthcare usage [27,79]. To our knowledge, these are the only examples in the current literature that draw empirical links between ‘group membership’, ‘community belonging’, ‘social support’, ‘loneliness’, and related health care usage.

### 4.2. Programme Delivery

Nearly all of the social prescribing programmes covered in this literature were delivered based on considerations that pilot programmes typically confront, with participants chosen according to need, funding restrictions, and accessibility. Such circumstances make it difficult to deliver programmes according to an externally valid research design that yields insights applicable outside of the pilot participant group. The resultant evidence is then often either so case specific or so insufficient in rigor as to be an unreliable source of guidance in planning or delivering programmes elsewhere.

With the expansion of funding, support, and requirements for social prescribing, we recommend that delivery be more carefully designed to enable comparisons between treated and untreated groups. If social prescribing is intended to be eventually offered to all residents in an area, a phased rollout would enable data collection for people before and after they engage with the programme, and comparison among participants that started and finished at different times would approximate a treated/untreated analysis. If a programme will be first administered via health care facilities and then expanded to other referral pathways, or first offered to one age group before expanded to others, a similar phased approach to collecting data could be used. In such cases, collecting and analysing information on other potential drivers of well-being, such as demographic and health information, is a recommended way to distinguish the effects of the social prescribing programme from other environmental or individual attributes.

Improving both design and delivery as recommended will reap many benefits. On practical grounds, sound conceptual development helps make the case for programme continuation and funding because decision makers can more readily understand how inputs lead to outcomes. Operationally, outcome and impact evaluation become straightforward because clearly linking outcomes of interest to measurement essentially designs the programme evaluation at the same time. Finally, conceptual and methodological clarity and programme delivery contribute to analytical robustness, which means findings and learnings can be useful in contributing to an evidence base for further decision making about social prescribing around the world.

### 4.3. COVID-19

In 2005, the World Health Organisation (WHO) adopted a resolution to support member states to actively engage in building capacity to respond to emerging public health issues [52]. Since then, the international community has faced numerous health crises, most recently the coronavirus (COVID-19) pandemic that began in 2020. The pandemic has caused social and economic disruptions and brought the issues of loneliness and social isolation to the forefront of public health agendas. COVID-19 has tested individual, systemic, and community health systems and resilience around the world.

Because our review concludes with studies published on or before 6 October 2020, approximately 10 months after the emergence of COVID-19, there was not time for a study on COVID-19 and social prescribing to be conducted, evaluated, and published by the time of our search. Nonetheless, there is evidence that social prescribing has been widely used to try to combat effects of the pandemic. Some studies emphasise the importance of keeping in touch and suggest that social prescribing programmes and services have a significant role in combating the pandemic’s consequences on health [2]. In a recent testimony at the Select Committee on COVID-19, Olivia Field, Head of Health and Resilience Policy at the British Red Cross, said that those who are feeling lonely “always” or “often” are likely to be less able to cope with challenges that arise, and that COVID-19 has made lonely individuals feel even more lonely [96]. Similar findings were reported by UCL COVID-19 Social Study researchers [97].

Given the negative impacts of COVID-19 on health, in particular loneliness, well-being, and a sense of belonging, structures and strategies to promote virtual connections are of utmost importance. Online groups that allow people to continue their hobbies such as signing, reading, or touring museums are just some of the strategies implemented via social prescribing services. One social prescribing programme, Connected Communities, is utilising virtual technologies such as tablets (Grandpads) and mobile service units (vehicles) to reach lonely and socially isolated individuals in rural areas [98,99,100]. We expect to see multiple studies on social prescribing and the pandemic to emerge in the coming months.

### 4.4. Strengths and Limitations

To our knowledge, ours is the first systematic review to address the impact of social prescribing programmes on four related, yet distinct aspects of individual health: loneliness, social isolation, well-being, and connectedness. We are also the first to our knowledge to review the impact of social prescribing programmes at three levels of analysis: individual, system, and community. We identify areas where connections between social prescribing and impact are well documented (loneliness; well-being; health care) and areas where evidence of impact needs further development (social isolation; connectedness; social care; community). In assessing study quality, we differentiate between those that provide evidence of change and those that establish causal links between an observed change and a social prescribing intervention. We thereby synthesise the current literature’s contributions to an understanding of social prescribing and its impacts.

We have sought to minimize the limitations of this systematic review by searching a wide range of published articles and grey literature reports spanning the period over 20 years, without geographical limitations. Our review also shows that social prescribing is being considered beyond the UK, with programmes and studies spreading around the world and across various disciplines. We do acknowledge the potential that we have missed some studies based on our search terminology or English language restriction. We have made every effort to be as inclusive as possible by searching nine databases using six versions of the search string and an extensive search of grey literature to minimize any potential bias. We maintain that any studies we might have missed would not likely provide findings reliable or conclusive enough to substantively alter our conclusions or recommendations.

We also acknowledge substantial limitations in our ability to make any conclusions regarding the impact of social prescribing or its potential as an innovative and financially sustainable means to deliver or manage preventive and public health. The number of studies that can attribute a change in outcomes to a related social prescribing intervention is low, and evidence of the impact of social prescribing on social care is lacking. Only more work to develop and utilise validated and comparable outcome measures will change the ability to draw conclusions and fully understand the overall impact of social prescribing.

## 5. Conclusions

Social prescribing is recognised as a vital resource for its potential to positively impact health, enhance individual and community assets to address consequences of issues such as loneliness and social isolation, reduce vulnerabilities, and build social support among individuals during the pandemic and beyond [101]. The most recent report by the UK Government on their Loneliness Strategy efforts to reduce loneliness specifies social prescribing as a useful and needed model for providing welfare checks and practical support for the world’s loneliest and most isolated individuals [12].

Levels of loneliness, isolation, well-being, and connectedness were considered a threat to public health even before the COVID-19 pandemic [8,102]. Finding ways to combat loneliness and social isolation has become a central focus of governmental and community organisations in the United Kingdom (UK) and is rapidly becoming pertinent across the rest of the international community. Overall, there is a positive response to the social prescribing initiatives, from participants, from the health and social care staff, and from community actors involved in programme delivery. Yet improvements are needed in clarifying and measuring outcomes, particularly in terms of differentiating concepts from each other and from similar concepts. The studies that evidence how improvements changes in loneliness, well-being, isolation, and connectedness help individuals engage with others, gain independence, gain better control of their health, and better understand available resources and how to utilise these, provide the strongest theoretical basis from which to link these elements to financially sustainable preventive and public health. Creating and maintaining these positive impacts, and gaining new skills, insights, and resources to improve one’s health, *should* position individuals to contribute to their own health and the health of those around them, take demand off of overburdened health and social care systems, and contribute to greater community productivity. Whether or not these processes *do* occur remains to be investigated.

As an innovative approach to managing health and social care, social prescribing has the potential to offer both preventive and acute benefits to individual health, system-level management, and community-level well-being. The best thing about social prescribing is also its greatest weakness: *potential*. It seems that social prescribing has the potential to help address nearly any problem related to individual health and well-being; over-burdened health and social care systems; and community connectedness, resilience, and productivity. Yet in recognising the breadth of possibilities social prescribing can address, research into social prescribing efficacy becomes equally broad, considering divergent and incomparable outcomes, measures, and programme designs. Until evaluations of social prescribing programmes become more standardised and comparable, the vast potential of social prescribing to cause change both increases the reasons to implement the approach and weakens the argument for doing so.

While the studies that effectively demonstrate and evidence impact of their programmes are useful and informative, others that are less successful in providing such evidence can also contribute to our understanding on how social prescribing works and what can be done to better assess its impact. This concern is particularly relevant when it comes to social isolation, the issue most commonly cited as a reason for participation in social prescribing programmes, yet also the issue least likely to have sufficient conceptual or methodological clarity when evaluated. Importantly, our work reveals a gap in the current social prescribing and public health literature on establishing the links that exist between various health and well-being outcomes and ways to account for these related, yet distinct, phenomena.

Our review highlights an urgent need to develop and establish guidelines to assess the impact of social prescribing at the community level, because it is precisely at the community level that preventive and public health innovations are most needed. Given that social prescribing is a community-based initiative, with community resources being the central focus of delivery, more effort is necessary to establish impacts of these programmes on structures that play a fundamental role in its implementation. Research into social prescribing would also benefit from greater acknowledgement and consideration of the complexities of health and its implications for designing and implementing person-centred programmes such as social prescribing. The evidence needed to assess the impact ranges from contextual factors (target population, needs, referral sources) to individual and community health and well-being measures, and sectoral conditions, including effects on the voluntary, public, and corporate sectors.

Initiatives that address underlying vulnerabilities at the individual, system, and community level are rapidly expanding, and we look forward to new releases of evidence and impact evaluations every day. As studies and evidence are implemented and updated, we urge evaluators to consider improvements in loneliness, isolation, well-being, and connectedness to be not just an end in themselves, and not just a means by which to reduce demands on the health and social care system, but also as a step closer to successful achievement of financial sustainable universal health.

## Figures and Tables

**Table 1:** Summary Table of 51 articles that evaluate social prescribing programmes according to individual-level outcomes.

Impact Concept	Concept Measure (Number of Studies Utilising the Measure)	Evidence of Change ^#^	Means of Causal Inference ^##^
Loneliness (14)	UCLA (7) Delphi Method (1) Hawthorne Friendship Scale (1) Scale developed for a study purpose (1) Unspecified measure (4)	4 [27,37,79,85] ^†^ 1 [80]	Serial mediation analysis, *Coeff* = (−0.23), *p* = 0.003 [27] ^†^ Serial mediation analysis, *Coeff* = (−0.31), *p* = 0.0001 [79] ^†^
Social Isolation (20)	Conf. with loneliness (3) Conf. with connectedness (1) Interview quotations (2) Thematic analysis (2) Hawthorne Friendship Scale (1) Duke-UNC (1) Adult Social Care/Public Outcome (1) Unspecified measure (9)	2 [60,66] ^†^ 1 [76]	Randomised controlled trial (RCT), *non sig.* mean between group diff., DUKE-UNC: confidant support (−0.9), *p* = 0.221) and affective support (−0.3), *p* = 0.594 [60] ^†^
Well-being (42)	WEMWBS (14) ONS4 (2) UCL Museum well-being (2) Scale developed for a study purpose (8) Thematic analysis (4) The Kessler Psychological Distress Scale K10 and The World Health Organization Quality of Life (1) Medical outcome profile (MYMOP) (1) Investigating Choice Experiments for the Preferences of Older People Capability Measure for Adults (ICECAP-A) (1) Unspecified measure (9)	15 [26,37,38,44,55,57,59,60,61,63,64,65,66,67,68,69,70,71,73,75,76,77,78,80,81,82,83,85] ^†^ 13 [26,55,64,65,67,68,69,73,75,76,78,80,83]	Randomised controlled trial (RCT), *sig.* mean score between group diff. (−1.9), *p* = 0.002) [60] ^†^ Randomised controlled trial (RCT), mean between-group diff., the WEMWBS score at 3-month follow-up 4.6 (−1.7 to 10.8), and 1.9 (95% CI −4.6 to 8.4) at 6 months. [81] ^†^ Cluster-RCT, *Coeff* = (–0.011), *p* = 0.411 [57] ^†^
Connectedness (14)	Thematic analysis (4) Scale developed for a study purpose (2) Adult Social Care/Public Outcome (1) Campaign to End Loneliness (1) Interview quotations (1) Revised UCLA loneliness scale (1) Unspecified measure (4)	3 [27,79,82] ^†^	Serial mediation analysis, community belonging *(Coeff* = 0.09, *p* = 0.01); social support (*Coeff* = 0.33, *p* < 0.001) [27] ^†^ Serial mediation analysis, *Coeff* = 0.09, *p* = 0.01 [79] ^†^

# Studies showing statistically significant change between pre- and post-measures for a target population/group of interest, with these measures clearly defined and connected to key outcomes. Detailed findings reported in Table S1. **^##^** Studies implementing a research design or can claim a causal link between the social prescribing intervention and the changes they find due to research design (RCT), statistical matching, or other rigorous means of causal inference. ^†^ Studies published in peer-reviewed outlets.

**Table 2:** Summary table of 22 articles that evaluate social prescribing programmes according to system-level outcomes.

Impact Concept	Concept Measure (Number of Studies Utilising the Measure)	Evidence of Change ^#^	Means of Causal Inference ^##^
Health Care (21)	GP care visits (9) A&E attendance (9) Inpatient admissions (7) Cost savings (5) Outpatient appointments (3) Referral pathways effectiveness (2) Hospital discharge (1) Mental health services discharge (1) Mental health services visits (1)	7 [60,61,63,66,72,79] ^†^ 14 [26,55,58,64,67,68,69,73,74,75,76,78,83,95]	Randomised controlled trial (RCT), no diff. between groups, mean = 4.4 [60] ^†^ Serial mediation analysis, *Coeff* = 1.41, *p* = 0.002 [79] ^†^
Social Care (3)	Contact with social care worker (1) Cost savings (1) Impact on care home services (1)	1 [63] ^†^ 1 [55]	

^#^ Studies showing statistically significant change between pre- and post-measures for a target population/group of interest, with these measures clearly defined and connected to key outcomes. **^##^** Studies implementing a research design or can claim a causal link between the social prescribing intervention and the changes they find due to research design (RCT), statistical matching, or other rigorous means of causal inference. ^†^ Studies published in peer-reviewed outlets.

**Table 3:** Summary table of 18 articles that evaluate social prescribing programmes according to community-level outcomes.

Impact Concept	Concept Measure (Number of Studies Utilising the Measure)	Evidence of change *	Means of Causal Inference ^##^
Community Resources (14)	Societal Return on Investment (SROI) (4) Building capacity in a community (4) VCSE improvements and funding access (3) Increase in usage of community resources: groups, services, activities (1) Increase in the number of community activities and groups available in a community/make services more available (1) Social prescribing volunteers securing paid employment (2) Integrating services and creating infrastructure to support service delivery (2)	1 [44] ^†^ 7 [26,55,58,67,73,76,80]	
Community Connectedness (3)	Community belonging (2) Promoting value of social connection (1)	1 [27,79] 1 [69]	Serial mediation analysis, community belonging *Coeff* = 0.09, *p* = 0.01 [79] Serial mediation analysis, community belonging *Coeff* = 0.09, *p* = 0.01; social support *Coeff* = 0.33, *p* < 0.001 [27]

^#^ Studies showing statistically significant change between pre- and post-measures for a target population/group of interest, with these measures clearly defined and connected to key outcomes. **^##^** Studies implementing a research design or can claim a causal link between the social prescribing intervention and the changes they find due to research design (RCT), statistical matching, or other rigorous means of causal inference. ^†^ Studies published in peer-reviewed outlets.

## Data Availability

Data is contained within the article or the supplementary material.

## Supplementary Materials

The following are available online at https://www.mdpi.com/article/10.3390/ijerph18105276/s1, Table S1: Detailed overview social prescribing programme evaluations designed to address loneliness, social isolation, well-being, and connectedness across the globe (2000–2020).
