# Supplementary material for: Can Social Prescribing Foster Individual and Community Well-Being? A Systematic Review of the Evidence
Source: Int J Environ Res Public Health. 2021 May 15;18(10):5276. doi: 10.3390/ijerph18105276 (PMC8156788; doi:10.3390/ijerph18105276)
Supplement: Supplementary file 1 [file ijerph-18-05276-s001.zip › ijerph-1148917-SI.pdf]

**Table S1.** Summary Table of the systematic review of social prescribing programmes designed to address loneliness, social isolation, connectedness and well-being across the globe, during 2000-2020 period. Supplementary Table S1 provides detailed information on programmes.

| Author and programme | Issue/Measures/Design | Evidence and Impact | | |
|---|---|---|---|---|
| | | **Person** | **System** | **Community** |
| **Programme name:** 'CLICK into Activity' programme.[1]<br><br>**Location:** South West England, UK.<br><br>**Evaluated by:** Bird, Biddle and Powell, University of the West of England. | **Loneliness:** Not assessed.<br><br>**Social isolation:** Measure not specified.<br><br>**Well-being**: changes in Warwick-Edinburgh Mental Well-being Scale (WEMWBS).<br><br>**Connectedness:** Not assessed.<br><br>Interviews, survey, pre/post comparisons. | **Interview** evidence used to conclude reduction in social isolation.<br><br>**WEMWBS mean scores** statistically significant (stat.sig.) increase at 3, 6, 12-month follow-ups (Coeff=5.56, p<0.001). | Not assessed. | Not assessed. |
| **Programme name:** Fit for the Future, Age UK[2]<br><br>**Location:** Blackburn/Darwen; Cheshire East; Exeter; Leeds; Hillingdon; Lancashire; Newcastle; Nottingham/ Nottinghamshire; Rotherham; Warwickshire; West-Cumbria, UK. | **Loneliness, Social Isolation, Connectedness** used interchangeably. Revised UCLA loneliness scale used to assess all three (Hughes et al, 2004).[3] Lacking conceptual and measures clarity (see Appendix 1, Table 2).<br><br>**Well-being**: WEMWBS.<br><br>Surveys, pre and post comparisons. Case studies and interviews. | Reduction in loneliness (feel less lonely) and social isolation (feel less isolated), showing stat.sig. change in mean scores across timepoints 1-2 and 1-3. Conceptual and measurement clarity needed to establish the impact regarding each issue in this report. ¼ of participants report improvements in connectedness.<br><br>**WEMWBS mean scores** stat.sig. change between 1-3 timepoints T1=26.91 (4.690) and T3= 27.44 (4.781), p<.001. A small (not sig.) decrease in well-being scores between 2- 3 timepoints. | No statistically significant impact. | 40 % of volunteers reported securing paid employment following their volunteering in the programme – a benefit that impacts larger community. |

| | | | | |
|---|---|---|---|---|
| **Evaluated by:** Wigfield and colleagues, University of Leeds | | | | |
| **Programme name:** Amalthea Project[4]<br><br>**Location:** 26 general practices in the county of Avon, UK<br><br>**Evaluated by:** Grant and colleagues, University of Bristol. | **Loneliness:** Not assessed.<br><br>**Social Isolation:** Duke-UNC functional social support questionnaire<br><br>**Well-being:** Psychological well-being (assessed with the hospital anxiety and depression scale).<br><br>**Connectedness:** Not assessed.<br><br>Randomised control trial, pre/post study comparisons (1 and 4 months follow-up), survey. | **Duke-UNC scale survey responses** showed no stat.sig. difference between group that received the intervention (Amalthea project) and the control group (confidant support mean scores (-0.9), p=0.221) and affective support (-0.3), p=0.594)<br><br>**Psychological well-being** measures a stat.sig. difference between the control and intervention group ((-1.9), p=0.002)). Also stat.sig. change in mean scores between baseline and follow-up timepoints for the intervention group. | No statistically significant difference between groups, equal numbers of contacts with primary care mean= (4.4). The total cost of healthcare was significantly greater in the intervention group, having more mental health prescriptions, which could partly explain the observed costs. Even though this particular intervention did not result in overall health care savings, the intervention group which received more prescriptions reported a reduction in anxiety, which was beneficial in terms of overall individual health. | Not assessed. |

| | | | | |
|---|---|---|---|---|
| **Programme name:** Ways to Wellness[5]<br><br>**Location:** an inner-city area of high socioeconomic deprivation in the west of Newcastle upon Tyne, UK<br><br>**Evaluated by**: Moffatt and colleagues at the Newcastle University | **Loneliness:** Not assessed.<br><br>**Social Isolation:** A self-report during the interview-participants described or reported on the feeling of social isolation in their own words.<br><br>**Well-being:** measure not specified.<br><br>**Connectedness:** Not assessed.<br><br>Semi-structured interviews. Thematic analysis. | The report concludes that the programme offered opportunities for activities, which allowed people to meet and socialise in the community, which resulted in reduced social isolation, improvements in mental well-being, self-confidence and self-esteem. | Not assessed. | Not assessed. |
| **Programme name:** Ways to Wellness (WtW).[6] Follow-up study to the Moffatt et al 2017 study[5]<br><br>**Location:** Newcastle upon Tyne, UK<br><br>**Evaluated by:** Wildman and colleagues at the Newcastle University. | **Loneliness:** Not assessed.<br><br>**Social Isolation:** Not clear how it was assessed.<br><br>**Well-being**: Not assessed.<br><br>**Connectedness:** Not assessed.<br><br>Semi-structured interviews. | The report concludes that 1-2 years after the programme completion the participants report feeling less socially isolated and more engaged in community activities. | Not assessed. | Not assessed. |
| **Programme name:** The Glasgow Deep End Links Worker Programme[7]<br><br>**Location:** 15 Glasgow general practices serving patients living in some of | **Loneliness:** Not assessed.<br><br>**Social isolation:** Not assessed.<br><br>**Well-being:** Capability-based measure of well-being: Investigating Choice Experiments for the Preferences of Older People | No significant improvement in capability-based measure of well-being (Coeff=(–0.011), p=.411)). | Not assessed. | Not assessed, observation made that the usage of community resources increased with increase of contacts between the participants and |

| | | | | |
|---|---|---|---|---|
| the most deprived areas in Scotland.<br><br>**Evaluated by:** Mercer and colleagues at the University of Edinburgh and University of Glasgow. | Capability Measure for Adults (ICECAP-A).<br><br>**Connectedness:** Not assessed.<br><br>A quasi-experimental cluster-randomised controlled trial (RCT). Pre/post comparisons with outcomes measured at baseline and at 9 months of the participation. | | | programme link workers, with a slight decline after 4th meeting. |
| **Programme name:** The Glasgow Deep End Links Worker Programme [8]<br><br>**Location:** in areas of high socioeconomic deprivation in Glasgow, Scotland.<br><br>**Evaluated by:** Hanlon and colleagues at the University of Glasgow, UK. | **Loneliness:** Not assessed.<br><br>**Social isolation:** Not assessed.<br><br>**Well-being:** assessed using self-Determination Theory: autonomy (a sense of control over one's own activities and behaviours), competence (a sense of ability to influence outcomes) and relatedness (a sense of connection/interaction with others).<br><br>**Connectedness:** Not assessed.<br><br>Semi-structured interviews. | **Self-Determination theoretical framework** used to establish that: 6 participants reported moderate to major positive improvements in their overall well-being, 2 described slight improvements and 4 no overall change. | Not assessed. | Participants report greater participation in community activities and groups following their participation in the programme. |
| **Programme name:** Dance to Health[9]<br><br>**Location:** Cheshire, Oxfordshire, Norfolk, South Wales, | **Loneliness:** No measures specified.<br><br>**Social isolation:** No measures specified.<br><br>**Well-being:** No measures specified. | 94.7% participants felt Dance to Health has improved their mental well-being. The report claims that there are statistically significant changes in the following areas: feeling calm & relaxed, feeling confident, feeling a reduced sense of loneliness & | **Cost savings** of over £149m over a 2-year period, of which £120m is a potential cost saving for the NHS. There was also a 15% | Societal Return on Investment (ROI) estimates, Dance to Health has a potential ROI of £2.37 for every £1 invested, indicating |

| | | | | |
|---|---|---|---|---|
| Birmingham, Sheffield, UK.<br><br>**Evaluated by:** Sport Industry Research Centre (SIRC) at Sheffield Hallam University | **Connectedness:** Not assessed.<br><br>Survey, Pre/post programme comparisons. | isolation, feeling an increased sense of independence. | reduction in the A&E visits. | there is a positive return of £1.37. |
| **Programme name:** No Name, just social prescribing programme[10]<br><br>**Location:** London Borough of City and Hackney; a wide range of socio-economic deprivation and affluence.<br><br>**Evaluated by:** Carnes and colleagues at the Queen Mary University of London, University of Applied Sciences Western Switzerland, University of East London, and City and Hackney Clinical Commissioning Group. | **Loneliness:** Not assessed.<br><br>**Social isolation:** Measure not specified.<br><br>**Well-being:** Medical outcome profile (MYMOP), developed by Paterson 1996 study[11], 4-scale questionnaire which has one question on general well-being.<br><br>**Connectedness:** Not assessed.<br><br>Semi-structured interviews, baseline and 8-month follow-up. | No statistically significant effects on participants' general mental health and well-being (Coeff=(-0.013), p=0.035. No mention of the impact on social isolation. | Statistically significant drop in median GP consultation rate from 8.3 to 7.3, however, the authors conclude that these changes may reflect regression to the mean rather than changes related to the intervention. | Not assessed, observation made that a total of eighty-two community organisations were used in the delivery of the service, no further assessment on the impact of the programme on community. |
| **Programme name:** Arts on Prescription [12] | **Loneliness:** Not assessed.<br><br>**Social isolation:** Not assessed. | **WEMWBS scores** statistically significant increase between baseline and post program (mean increase of 7.25, 95% CI: 5.39–9.11, t = | Not assessed. | Not assessed, observation made that the participants |

| | | | | |
|---|---|---|---|---|
| **Location:** Sydney, Australia<br><br>**Evaluated by:** Poulos and colleagues at School of Public Health and Community Medicine, University of New South Wales, Australia, Hammond Care in Sydney, a General practitioner in Gloucestershire, and Sidney De Haan Research Centre for Arts and Health, Canterbury Christ Church University, UK. | **Well-being:** WEMWBS<br><br>**Connectedness:** Not assessed.<br><br>Survey, focus groups and interviews. Pre/post programme comparisons. | 7.78, df = 63, $p < 0.001$). The programme had a positive impact on participants' well-being. | | reported new friendships and meetings outside the programme. |
| **Programme name:** Active Lives[13]<br><br>**Location:** two Age UK Lancashire centres and rural locations.<br><br>**Evaluated by:** Bell and colleagues at Faculty of Health and Social Care at Edge Hill University and Liverpool Business School, Liverpool John Moores University, and | **Loneliness:** Not assessed.<br><br>**Social isolation:** Not assessed.<br><br>**Well-being:** A measure of well-being used in Bowling (2005)[14], without providing additional information on the measure or the definition.<br><br>**Connectedness:** Not assessed.<br><br>Focus groups and surveys. Pre/post-study comparisons. | 83% - 87 % of participants reported that attending the programme improved their well-being. | Not assessed. | Not assessed, observation made that the participants more engaged in their community by attending community groups. |

| | | | | |
|---|---|---|---|---|
| in partnership with Age UK Lancashire. | | | | |
| **Programme name:** 'Holistic' link-workers service/Well-being Coordination[15]<br><br>**Location:** South Devon<br><br>*Rare example of a study taking into account impact on social care services.<br><br>**Evaluated by:** Elston and colleagues | **Loneliness:** Not assessed.<br><br>**Social isolation:** Not assessed.<br><br>**Well-being:** WEMWBS<br><br>**Connectedness:** Not assessed.<br><br>Surveys. Pre/post-study comparisons. | **WEMWBS scores** showed a mean increase of 20.3% (P = 0.000), with a meaningful change (≥5 points) in 54 people (62.9%). | GP usage data not analysed, poor quality. Contrary to the expectations, there was an increase in the overall costs to the health (81.2% share of the total costs) and social care services. Increase was due to hospitalizations and social care costs increase due to a participant passing away (>£30,000). | Not assessed, observation made that there was an overall increase in community services following the programme implementation. |
| **Programme name:** Rotherham Mental Health Social Prescribing Service[16]<br><br>**Location:** Rotherham<br><br>**Evaluated by:** Dayson and Bennett at the Centre for Regional Economic and Social Research at the Sheffield Hallam University | **Loneliness:** Not assessed.<br><br>**Social isolation:** Not assessed.<br><br>**Well-being:** a tool designed to measure 8 aspects of personal, social and emotional well-being: feeling positive, lifestyle, looking after yourself, managing symptoms, work and volunteer activities, money, where you live, family and friends.<br><br>**Connectedness:** Not assessed.<br><br>Pre/post programme comparisons. | **Mean score** increases in 'work, volunteering and social groups', 'feeling positive', 'lifestyle' and 'managing symptoms' well-being outcomes. Those who scored low on the baseline score for all of the 8 aspects of the well-being had greater improvement in the mean score for each outcome when compared to scores for the rest of the sample. | Positive impact on mental health service usage: **85 participants discharged from mental health services** (54 % of those eligible for discharge review). Some have been using the mental health services for 5-20 years without being discharged successfully until their participation in the programme. | Not assessed, observation made that the participants that found employment (10); training and education (48); volunteering (38); acted to improve physical health (59); accessed peer-support opportunities (59); and voluntary and community org. (84). |

| | | | | |
|---|---|---|---|---|
| **Programme name:** The Rotherham Social Prescribing Service for People with Long-term Conditions.[17],[18]<br><br>The 2017 and 2020 report are combined into one section as it relates to the same programme and the participants<br><br>**Location:** Rotherham<br><br>**Evaluated by:** Dayson and Damm at the Centre for Regional Economic and Social Research at the Sheffield Hallam University | **Loneliness:** Not assessed.<br><br>**Social isolation:** Not assessed.<br><br>**Well-being:** a tool designed to measure 8 aspects of personal, social and emotional well-being: feeling positive, lifestyle, looking after yourself, managing symptoms, work and volunteer activities, money, where you live, family and friends.<br><br>**Connectedness:** Not assessed.<br><br>Pre/post programme comparisons. Spans 2012-2018 time period, with results divided in periods. | 82% of participants experienced positive change in at least one of the 8 areas of well-being. Feeling positive, money and work category the most consistent improvements during September 2012-2014.<br><br>35% feeling more positive (65% those who scored low on this dimension at the start), 26% making progress in their lifestyle, 23% more able to look after themselves, 23% more able to manage their symptoms, 46% making progress in finding work and volunteering, 28% more progress with money, 24% with where they live, 19% progress with family and friends, 2014/2015 period.<br><br>47% feeling more positive, 32% making progress in their lifestyle, 20% being more able to look after themselves, 28% more able to manage their symptoms, 46% making progress in finding work and volunteer activities, 30% more progress with money, 17% with where they live, and 17% progress with family and friends, 04/2016- 03/2018. | **Non-elective inpatient** spells reduced by 11% and **A&E attendances** reduced by 17%, 2012-2014.<br><br>**Cost avoidance** to the NHS to be close to £647,000, with an initial return on investment of 35 pence for each pound (£1) invested, 2012-2016.<br><br>Interestingly, during the 2016-2018 period there was a slight increase in the number and the cost of inpatient spell and A&E attendances. | The programme success has helped other organisations to build capacity through additional funds being offered by Voluntary Action Rotherham and other funders. The programme had a positive impact on community, developing and promoting social action and volunteering and promoting the value of social connection. |
| **Programme name:** No Name[19]<br><br>**Location:** most likely in a community in Yorkshire, location not mentioned. | **Loneliness:** Not assessed.<br><br>**Social isolation:** Social Networks measure used to determine social connectedness.<br><br>**Well-being:** WEMWBS | **WEMWBS scores** overall increase: 265 (77.5%) participants have improved well-being score from baseline to post stage. Also, 58 (17%) had a decrease in score and 19 (5.6%) had no overall change. Improvements in well-being were also observed in the **interviews** with individuals describing feelings of optimism and a more | 53.3% of participants reported using GP services about the same, no change. Rest of the participants reported reduction in **GP usage**: 27.2% of participants had used | Not assessed. |

| | | | | |
|---|---|---|---|---|
| **Evaluated by:** Woodall and colleagues at the Leeds Beckett University, University of York, Leeds City Council, Connect for Health Hillside and NHS Leeds CCG's Partnership, all in the UK. | **Connectedness:** Social Networks measure used to determine social connectedness.<br><br>**Social Networks** (Isolation and/or Connectedness): Campaign to End Loneliness Measurement Tool – a short 3-item scale – which examines social networks.<br><br>The report does not differentiate between social isolation and social connectedness measures.<br><br>Surveys and interviews, pre/post programme comparisons. | positive outlook as a result of being referred to the service.<br><br>**Interview analysis** showed improved sense of social connectedness as a result of engaging with the social prescribing service.<br><br>Around half of the participants 155/306 (50.7%) had an improved 'Social Networks' score from baseline to post stage; 76 (24.8%) had a decrease in score; and 75 (24.5%) had no overall change. | services less, with 5.5% visiting 'a lot less' and 21.7% a 'bit less'. | |
| **Programme name:** Gloucestershire CCG's social prescribing[20]<br><br>**Location:** Gloucestershire<br><br>**Evaluated by:** Kimberlee at the University of the West of England Bristol (UWE). | **Loneliness:** Not assessed.<br><br>**Social isolation:** Not assessed.<br><br>**Well-being:** WEMWBS<br><br>**Connectedness:** Not assessed.<br><br>Surveys and interviews, pre/post programme comparisons. | **WEMWBS scores** stat.sig. increase baseline (M =18.51, SD 6.1) to follow up (M=22.37, SD 5.9) t (398) =-16.21. | ***GP visits*** declined by 21% in the six months after referral compared to six months before. Also, number of home visits declined by 26% and the number of telephone calls by 6%. ***A&E attendance*** declined by 23% in the six months after referral compared to the six months before. | Improvements in community engagement. Most of 49 VCSE organisations were satisfied with the programme, reporting that the participation in the programme has helped them raise awareness of their work and mission. |
| **Programme name:** 8 different programmes across Wessex were | **Loneliness:** Not assessed.<br><br>**Social isolation:** Not assessed. | Improvement in well-being score, with the greatest change in life satisfaction, with overall **mean ONS score** of 66 after the | 31% reduction the ***A&E*** attendance and 32 % in ***Inpatient Admissions*** for the Mid Hampshire | Not assessed. |

| | | | | |
|---|---|---|---|---|
| evaluated in one report.[21]<br><br>**Location:** Wessex<br><br>**Evaluated by:** Wessex Academic Health Science Network (WAHSN) and R-Outcomes | **Well-being:** ONS4.<br><br>**Connectedness:** Not assessed.<br><br>Survey, pre/post programme comparisons. | programme participation, in comparison to 58 at the start of the programme. | Healthcare system. For the Eastleigh Southern Parishes healthcare system reduction in A&E attendance of 50% and 32% in ***Inpatient Admissions***. | |
| **Programme name:** Community Navigation[22]<br><br>**Location:** not specified, most likely Brighton and Hove.<br><br>**Evaluated by: by** Farenden and colleagues at Impetus. Brighton & Hove Impetus developed the Community Navigation model in partnership with AUKBH and under the guidance of the lead partner Brighton & Hove Integrated Care Service (BICS). | **Loneliness:** Not assessed.<br><br>**Social isolation:** Not assessed.<br><br>**Well-being:** Two questions, 1) asked whether the patient was satisfied with the amount of time they spent with others as a result of participating in the programme 2) whether there had been an improvement in their general sense of well-being after seeing the Community Navigator.<br><br>**Connectedness:** Not assessed.<br><br>Surveys and interviews, pre/post programme comparisons. | 84% of participants experienced improvements in their sense of well-being. | The report estimates a net **cost saving** for GP services of £1365 per participant. The survey of GP staff showed that 68% of GP practice respondents thought that the programme was effective at reducing the amount of time patients attend the surgery with non-medical matters, whilst 19% did not know. | Contributed to the Brighton & Hove's Mental Health and Well-being Strategy. Health equality - increased access for people that would not otherwise be engaged with community activities/ services. Improved community cohesion by integrating services, with GPs being more aware of what local organisations can do to alleviate some of the pressures. Positive impact on building community social capital by providing volunteering opportunities and assistance for local |

| | | | | |
|---|---|---|---|---|
| | | | | people to enhance their skills and employment prospects. |
| **Programme name:** Voluntary Action Rotherham, coordinated by Sense.[23]<br><br>**Location:** Rotherham<br><br>**Evaluated by:** Vogelpoel and Jarrold at Sense, London, UK. | **Loneliness:** Not assessed.<br><br>**Social isolation**: measure not specified.<br><br>**Well-being:** WEMWBS<br><br>**Connectedness:** Not assessed.<br><br>Mixed methods: questionnaire completed at the first and last session; case studies and interviews with staff. Pre/post comparisons. | **Increase in overall mean well-being score**, from 41 to 47. The average well-being score in Scotland is 49.9, which points to the need for these types of programmes in this particular region, as the scores are lower than the general population. The study mentions score for Scotland even though the location of the programme is in Yorkshire. | Not assessed. | Not assessed, observation made that improvements in community connections – participants lobbying for the sessions to be extended. |
| **Programme name:** The Arts on Prescription[24]<br><br>**Location:** not clear, possibly Nottingham<br><br>**Evaluated by:** Stickley and Hui at the University of Nottingham, UK | **Loneliness:** Not assessed.<br><br>**Social isolation:** Not assessed.<br><br>**Well-being:** having a sense of purpose.<br><br>**Connectedness:** a sense of belonging to a group and acceptance.<br><br>A vignette used to review the interview transcripts.<br><br>Narrative inquiry, in-depth interviews, thematic analysis. | Several participants (no number) reported that the **programme gave them a sense of purpose, which is a component of well-being**.<br><br>The sense of confidence was greatly shaped by **being part of the group** while participating in the programme, which also improved their sense of belonging and acceptance**-improved connectedness.** | Not assessed. | Not assessed. |

| | | | | |
|---|---|---|---|---|
| **Programme name:** Healthy Connections Stewartry (HCS)[25]<br><br>**Location:** Dumfries and Galloway, South West Scotland.<br><br>**Evaluated by:** Whitelaw and colleagues at the University of Glasgow, Health & Well-being Specialist at the DG Health & Well-being NHS and Public Health Practitioner at the Health Improvement Team, NHS Dumfries and Galloway, UK. | **Loneliness:** Not assessed.<br><br>**Social isolation**: measure not specified.<br><br>**Well-being:** WEMWBS<br><br>**Connectedness:** Not assessed.<br><br>A case study design and 1–1 semi-structured interviews with the project steering group; the wider primary care team; and various community groups. | The community members, staff and individuals from the community groups associated with the programme, reported that greater inclusion improves people's well-being and that the HCS programme is well designed to address both, isolation and well-being related issues. No reports on the well-being scores. | The primary care staff saw the following benefits: the programme provided useful structures to streamline and simplify referrals of individuals to community services; holistic approach - more effective and sustainable health service. | Enabling community resources to be more accessible to the wider community. |
| **Programme name:** Not clear. Poster Abstract for the International Conference on Integrated Care, Utrecht, Netherlands.[26]<br><br>**Location:** Luton<br><br>**Evaluated by:** Pescheny and colleagues at the University of Bedfordshire. | **Loneliness:** Not assessed.<br><br>**Social isolation:** Not assessed.<br><br>**Well-being:** WEMWBS<br><br>**Connectedness:** Not assessed.<br><br>A mixed-methods study. Semi-structured interviews with participants, analysed using thematic | A significant improvement in participants' mental **well-being $t67 = 5.026$, $p= 0.00$ post intervention.** Qualitative findings also indicate improvements in well-being. | Not assessed. | Not assessed. |

| | | | | |
|---|---|---|---|---|
| | analysis. Survey responses to WEMWBS. Pre/post. | | | |
| **Programme name:** The British Red Cross (BRC), in collaboration and funded by the Co-op. [27]<br><br>**Location:** 37 locations across the UK<br><br>**Evaluated by:** Holding and colleagues at the University of Sheffield | **Loneliness:** UCLA loneliness scale.<br><br>**Social isolation:** Not assessed.<br><br>**Well-being**: Not Assessed.<br><br>**Connectedness:** Not assessed.<br><br>Semi-structured interviews with link workers and volunteers, 30- to 90-min duration. Thematic analysis, with research team meeting regularly to ensure the validity of the coding framework. Surveys. | The report provides views of the social prescribing staff on the impact of the programme. Staff members reported that a longer-term support was necessary in order for the social prescribing scheme to improve loneliness among participants. | Staff members reported that the referrals in some cases were not appropriate for the level of the support that they offer. As the success of the programme depend on the successful referral pathways, they suggest improvements in this aspect. | Gaps in community infrastructure created challenges for service delivery, highlighting the need for further commissioning of transport, community and befriending services alongside social prescribing. |
| **Programme name:** Activity Promotion Project (APP)[28]<br><br>**Location:** possibly London, UK<br><br>**Evaluated by:** Jacob and colleagues, place of work not clear. | **Loneliness:** Not assessed.<br><br>**Social isolation:** Not assessed.<br><br>**Well-being**: measure not specified.<br><br>**Connectedness:** Not assessed.<br><br>Survey, pre/post programme comparisons. | 97 % of the staff surveyed expressed said that patient's mood improved since the programme start, concluding that a programme had a positive impact on participants' well-being. | The percentage of patients (participants) resting in bed has declined, from 59% before to 49% during the intervention, indicating potential positive benefits of the programme for this particular hospital discharge process. | Not assessed. |
| **Programme name:** No name given[29] | **Loneliness:** UCLA 3-item Loneliness Scale.<br><br>**Social isolation:** Not assessed. | No significant differences in well-being ($M_B = 25.3(9.38)$, $M_F = 22.8(8.23)$, $t(11) = 1.145$, $p = 0.277$) and loneliness levels, however, loneliness scores ($M_B$ = | Not assessed. | Not assessed. |

| | | | | |
|---|---|---|---|---|
| **Location:** Sydney, Australia<br><br>**Evaluated by:** Aggar and colleagues at the Southern Cross University and The University of Sydney, Australia | **Well-being:** The Kessler Psychological Distress Scale K10 and The World Health Organisation Quality of Life (QoL).<br><br>**Connectedness:** Not assessed.<br><br>Surveys, pre/post programme comparisons. | 6.4(2.29), $M_F$ = 6.1(2.17), $t(10) = 0.412$, $p = 0.689$), did show a trend in a desired direction, decreasing over the course of the study. | | |
| **Programme name:** No name given[30]<br><br>**Location:** not specific, experts recruited from all over the UK.<br><br>**Evaluated by:** Leslie and colleagues at the Northumbria University. | **Loneliness:** Delphi method<br><br>**Social isolation:** Delphi method<br><br>**Well-being:** Not assessed.<br><br>**Connectedness:** Not assessed.<br><br>Delphi method, engaging experts (military veterans or work with) in the field of loneliness and social isolation to gain their understanding of the phenomena. Survey and thematic analysis. | The report concludes that social prescribing can play a positive role in the period of transition from military to civilian life, particularly in addressing issues of loneliness and social isolation. Social prescribing is viewed as a useful tool to help veterans integrate into the wider community and help link veterans to relevant community services. | Not assessed. | Not assessed. |
| **Programme name:** Self-Care, operated by Kensington and Chelsea Social Council (KCSC)[31]<br><br>**Location:** Kensington, Chelsea, the Queens Park and Paddington | **Loneliness:** Not assessed.<br><br>**Social isolation:** Not assessed.<br><br>**Well-being:** WEMWBS<br>Based on the note in Appendix section of the paper, p.45, WEMWBS is used to assess loneliness and social isolation in addition to well-being. It | The report finds a reduction in social isolation and loneliness and improvements in well-being. A slight reduction in social isolation, with 69% of participants reporting that they had enough people they personally felt close to in their lives after the service compared to 65% before. Overall improvements in well-being were noted in improved self-confidence, independence | 23-40% decrease in ***GP visits*** and 11.5% decrease in hospitalizations. The improvements in PAM scores were related to reduced usage of healthcare system, with | Based on the Social Return on Investment (SROI) estimates, the programme created an overall £2.80 social value for every £1 invested. Health services had the highest SROI of £4.30 |

| | | | | |
|---|---|---|---|---|
| areas of Westminster, UK.<br><br>**Evaluated by:** Envoy Partnership. | is not clear which WEMWBS items were used to measure loneliness or social isolation.<br><br>**Connectedness:** Not assessed.<br><br>Surveys and interviews, pre/post programme comparisons. | and dignity. The changes in the loneliness scores are not reported. | estimated monetary savings of £106,000. | created for every £1 invested. Patient health and well-being SROI value was £3.45 for every £1 invested. These findings indicate that the programme contributed to cost-saving and usage of a number of community resources. |
| **Programme name:** Social Prescribing Pilot[32]<br><br>**Location:** Rotherham, UK<br><br>**Evaluated by:** Dayson and Bashir at the Centre for Regional Economic and Social Research, Sheffield Hallam University | **Loneliness:** measure not specified.<br><br>**Social Isolation:** measure not specified.<br><br>**Well-being:** Not assessed.<br><br>**Connectedness:** Not assessed.<br><br>Interviews and case studies. Pre/post comparisons. | Participants report feeling like they belong more to a community and that they have enjoyed more social contact, with researchers drawing conclusions on reduction in loneliness and isolation. | ***A&E:*** 38% of participants report a reduction in attendance 12 months post-referral, 25% report reduction 6 months post-referral. ***Inpatient Admissions**:* 40% reduction 12 months post-referral, 24% 6 months post-referral. ***Outpatient admissions:*** 47% reduction 12 months post-referral, 30% 6 months post-referral. | Small organisations without previous access to NHS funding were able to access it for the first time, which enhanced their provision and improved their sustainability. |
| **Programme name:** Not reported[33]<br><br>**Location:** Unnamed local authority area, UK | **Loneliness:** measure not specified.<br><br>**Social Isolation:** measure not specified. | Quotations evidence a reduction in loneliness and social isolation.<br><br>In regards to well-being, 83% of participants reported making progress on at least one of | ***A&E***: 20% reduction in number of visits in 12-month post-participation period. ***Inpatient admissions:*** | Reports that unspecified number of participants became volunteers engaged in wider voluntary and |

| | | | | |
|---|---|---|---|---|
| **Evaluated by:** Dayson, Sheffield Hallam University | **Well-being:** an eight-item five-point scale, completed at two points in time: at referral and four months after. The report does not provide the name of the questionnaire used. The following well-being outcomes were identified: feeling positive, lifestyle, looking after yourself, managing symptoms, work, volunteering and social groups, money, where you live and family and friends.<br><br>**Connectedness:** Not assessed.<br><br>Surveys and interviews, pre/post programme comparisons. | the well-being outcome measures. Those with the lowest scores on well-being outcomes exhibited the greatest amount of change. | 21% reduction in the number of admissions in 12-month post-participation period. ***Outpatient appointments:*** 21% reduction in the number of admissions in 12-month post-participation period. | community activity once pilot concluded. Also, a number of local organisations receiving additional pump-priming grants as a result of their work on this programme. |
| **Programme name:** Doncaster Social Prescribing[34]<br><br>**Location:** Doncaster, UK<br><br>**Evaluated by:** Dayson and Bennett at the Centre for Regional Economic and Social Research, Sheffield Hallam University | **Loneliness:** Not assessed.<br><br>**Social isolation:** Not assessed.<br><br>**Well-being:** financial well-being – how well people are 'managing financially' - from 'finding it very difficult' to 'living comfortably'<br><br>**Connectedness:** “Social connectedness: a 'social isolation and loneliness' scale - from 'having as much social contact as I want' to 'having little social contact with people and feel socially isolated' | 19% increase in the number of participants having “enough social contact” when comparing baseline to follow-up scores. Participant report feeling less isolated or alone post-participation, “feeling like they had someone they could turn to”. The report concludes that these findings support evidence on the improvements in social connectedness. However, terms loneliness, social isolation and connectedness are used interchangeably.<br><br>Overall, there were improvements in financial well-being. | ***GP visits:*** 68% of participants report reduction in visits; 15% report increase; 17% no change. ***A&E:*** 7% report reduction; 1% report increase; 92% no change. ***Inpatient Admissions:*** 9% report reduction in stays; 3% increase; 90% no change. 5% of participants reported reduction in usage of *mental health services*, 5 % reported reduction | A non-specified number of volunteers have found employment since being involved the project. 88% report greater awareness of the services and support available. |

| | | | | |
|---|---|---|---|---|
| | - is used to provide a measure the amount and quality of social contact. The measure is based on the Adult Social Care and Public Health Outcome Framework (ASCOF/PHOF) indicator of social isolation and loneliness."<br><br>Pre and post-programme comparisons. Surveys. | | at the end of the programme participation. ***Social Care:*** 3% report reduction in contacts with social worker; 97% report no change (3% of sample reported having a contact with social services 3 months prior to start of the program).<br><br>For every £1 of health and social care funding spent supporting vulnerable people, the Social<br><br>Prescribing Service produced more than £10 of benefits in terms of better health, at least in the short term. | |
| **Programme name:** Wellspring Well-being Programme[35]<br><br>**Location:** Bristol, UK | **Loneliness** Hawthorne (2000) Friendship Scale and Wellspring Well-being Questionnaire | Three months after the start of their programme, the participants showed significant improvements on Hawthorne (2000) friendship scale scores which authors refer to as connectedness, but in fact is a | ***GP* visits:** 60% reduction in the GP attendance rates in the 12 months post intervention when | Enhancing community capacity: harnessing volunteers, beneficiaries returning to employment and |

| | | | | |
|---|---|---|---|---|
| **Evaluated by:** Kimberlee and colleagues. | **Social Isolation** Hawthorne (2000) Friendship Scale and Wellspring Well-being Questionnaire<br><br>**Well-being:** ONS4<br><br>**Connectedness:** not specified.<br><br>Connectedness, social isolation and loneliness are used interchangeably.<br><br>Pre/post; Interviews and questionnaires. | measure of social isolation—baseline (M=8.63, SD=6.01) to three months after (M=13.17, SD=4.28), t (69) = 5.62, p= < 0.001. Interviews with participants have shown that they are feeling like they belong more to a community and that they have enjoyed more social contact, with researchers drawing conclusions on reduction in loneliness (1 quote from participant) and social isolation. | compared to the 12 months prior to intervention. ***A&E:*** 38% of participants report a reduction in attendance 12 months post-referral, 25% report reduction 6 months post-referral. ***Inpatient Admissions****:* 40% reduction 12 months post-referral, 24% 6 months post-referral. ***Outpatient admissions:*** 47% reduction 12 months post-referral, 30% 6 months post-referral. Impact greater for participants referred to other funded services (48% reduction of inpatient admissions, 43% in A&E visits, 12 months post-referral). | training and resuming child care responsibilities. A Social Return On Investment (SROI) analysis showed that for every £1 invested in intervention, £2.90 of social value is created. |
| **Programme name:** Bristol Arts on Referral Alliance[36]<br><br>**Location:** Bristol, UK | **Loneliness:** Not assessed.<br><br>**Social isolation:** Not assessed.<br><br>**Well-being:** WEMWBS<br><br>**Connectedness:** Not assessed. | Well-being scores increased from 37.79 to 42.80, a mean increase of 5.01 units (3 units indicate 'meaningful change') during the first 12-week programme. For participants who returned to participate in second 12-week programme, well-being continued to improve, on average, rising from 39.93 to 44.66 (a mean increase of 4.73). During the | Not assessed. | Not assessed. |

| | | | | |
|---|---|---|---|---|
| **Evaluated by:** Holt, University of the West of England (UWE), UK. | Pre/post; surveys. | summer holidays, there was an interval period of 4.2 weeks (mean), with the report showing well-being scores decreasing during this period (e.g. from 42.80 to 39.93 (a difference of −2.87), however this decrease was not statistically significant ($t$ = 1.96, $p$ = .052). | | |
| **Programme name:** Hand in Hand (HiH)[37]<br><br>**Location:** Winchester, UK<br>**Evaluated by:** Wilkinson and colleagues, University of Winchester | **Loneliness:** Not assessed.<br><br>**Social isolation:** Not assessed.<br><br>**Well-being:** mention of a survey.<br><br>**Connectedness:** Not assessed.<br><br>* The evaluation period covers the preparation stage and very early implementation period. The outcomes and reported impact need further clarification.<br><br>The aim is to gather qualitative and quantitative data on participants' health and well-being status, basic data on services in Winchester, and the information to demonstrate value for money. The current report does not specify measure of well-being that will be used. The evaluation is based on 2 testimonials (case studies). | Early examples of 2 testimonials show that the programme is having a positive impact, with individuals who reported feeling lonely and socially isolated, becoming more confident to interact and engage with their community, thus, feeling less isolated. | Not assessed. | Not assessed. |

| | | | | |
|---|---|---|---|---|
| **Programme name:** Welzijn op Recept[38]<br><br>**Location:** Nieuwegein and other towns in the Netherlands<br><br>**Evaluated by:** Heijnders and Meijs, an independent researcher and a researcher at the Leids Universitair Medisch Centrum | **Loneliness:** Not assessed.<br><br>**Social isolation:** Not assessed.<br><br>**Well-being:** Well-being coaches used a variety of approaches to boost the participants' self-confidence, strength and self-resilience.<br><br>**Connectedness:** Not assessed.<br><br>Five overall themes were identified in the process of coding interviews: life events, referral and intake process, personal strength and responsibility, self- reliance and social activation/participation.<br><br>Interviews. | The participants reported the following benefits: gaining new experiences, meeting new people, exercising more and feeling good about it, having something to look forward to, regaining control, becoming more self-reliant, regaining perspective and experiencing improved health. The report provides support for increase in social participation and social contact which led to a sense of satisfaction, improving well-being. | Not assessed. | There is an interest to develop social activation approach that would enable participants to re-connect to their community and other community members through activities like being a volunteer in the community centre or participating in a social community activity, which some of the individuals have achieved (half of participants took up volunteering). |
| **Programme name:** Social Cure[39]<br><br>**Location:** Nottinghamshire, UK<br><br>**Evaluated by:** Wakefield and colleagues, Nottingham Trent University | **Loneliness** eight-item ULS-8 (Hays and DiMatteo, 1987), e.g. '*I lack companionship*' on a 1 (*not at all*) to 5 (*completely*) scale.<br><br>**Social isolation:** Not assessed.<br><br>**Well-being:** Not assessed.<br><br>**Connectedness:** Community belonging: ('*Thinking about this local community, the kind of place it is and the kind of people who live around here,* | There was a significant increase in the 'number of group memberships' during the participation in the programme (T0 and T1, F (1177) = 4.07, p = 0.04), with this change in group memberships between T0 and T1 being a positive predictor of community belonging (coeff= 0.09, SE = 0.04, t = 2.55, p = 0.01, LLCI =0.02, ULCI = 0.16), while community belonging T1 was a positive predictor of social support T1(coeff = 0.33, SE = 0.08, t = 4.32, p < 0.001,LLCI = 0.18, ULCI = 0.48), social support T1 was a negative predictor of loneliness T1 (coeff = −0.23, SE = 0.08,t = −2.99, p = 0.003, LLCI = | Not assessed. | Community belonging T1 was a positive predictor of social support T1(coeff = 0.33, SE = 0.08, t = 4.32, p < 0.001,LLCI = 0.18, ULCI = 0.48). |

| | | | | |
|---|---|---|---|---|
| | *would you say that you feel a sense of belonging to this local community?*').<br><br>Social support: four-item scale from Haslam et al. (2005). For example, '*Do you get the emotional support you need from other people?*') on a 1 (*not at all*) to 5 (*completely*) scale.<br><br>**Other measures:**<br><br>Number of group memberships: a list of 10 social groups to select from and a choice of 'not a member of any'.<br><br>All survey data were gathered in face-to-face meetings at the participants' GP surgeries. Pre/post comparisons. | −0.38,ULCI = −0.08), which itself was a negative predictor of QoL T1 (coeff = −0.06, SE = 0.02, t =−2.65, p = 0.009, LLCI = −0.11, ULCI = −0.02).<br><br>To our knowledge and to the knowledge of the authors of the Social Cure evaluation report, this is the first study of its kind that systematically links loneliness and community belonging. | | |
| **Programme name:** Museum on Prescription[40]<br><br>**Location:** London and Kent, UK[41]<br><br>**Evaluated by**: Todd and colleagues at the Canterbury Christ Church University and University College London. | **Loneliness:** R-UCLA Scale (Russell et al., 1980).<br><br>**Social isolation and Well-being:** the study cites the previous work (Chatterjee and Thomson, 2017) to point to the types of measures used to assess both at baseline and across the program, however, the previous work is not publicly available.<br><br>It appears that social isolation was observed in the terms of relating to others, particularly if evaluating self and others in a prejudiced manner | Participants report feeling less lonely, more able to develop meaningful connections and friendships, greater confidence, more mental stimulation and more feelings of happiness. The study claims that by offering opportunities for individuals to connect and engage and by providing them with meaningful support, the programme has helped enhance participants' sense of belonging, which in turn had a positive impact on well-being and reduction in social isolation. | Not assessed. | Not assessed. |

| | | | | |
|---|---|---|---|---|
| | contributed to social isolation. However, not included as it was not clearly specified.<br><br>Well-being it appears was assessed by WEMWBS and UCL Museums Well-being Measure – Older Adult.<br><br>**Connectedness:** Not assessed.<br><br>Case Study; Interviews, theory building using grounded theory analysis and inductive approach. | | | |
| **Programme name:** Artlift[42]<br><br>**Location:** Gloucestershire, UK.<br><br>**Evaluated by:** Redmond and colleagues at the University of Gloucestershire | **Loneliness:** Not assessed.<br><br>**Social isolation:** Not assessed.<br><br>**Well-being:** Not assessed.<br><br>**Connectedness:** The participants' responses to their views on outcomes, benefits and processes involved this particular programme were reviewed and the following themes were identified: being with others; being on my own; doing something for me; loosing oneself; threshold.<br><br>Thematic analysis. | Being with others was the most common benefit selected by the participants. The report concludes that the opportunity to meet and be with other people and be a part of the group has led to an improved sense of companionship and membership, which are viewed as aspects of connectedness. | Not assessed. | Not assessed. |
| **Programme name:** Integrated Plus. Dudley CVS[43] | **Loneliness / Social Isolation:** used interchangeably. Six indicators of social contact, no justification. | Number of people feeling lonely and without enough contact reduced by 46 % (87-46), p.32. Number of people feeling un- | ***GP visits:*** Of the 43 GP practices, 6 months post-programme 8 practices had an | Not assessed. |

| | | | | |
|---|---|---|---|---|
| **Location:** Dudley, UK.<br><br>**Evaluated by:** David Waterfall, Independent Evaluator | **Well-being:** Participants asked to rate their overall feeling of well-being from 1 to 5 (1 being low, 5 being high), at the start and the end of the programme.<br><br>**Connectedness:** Not assessed.<br><br>Case studies and surveys. Pre/post comparisons. | lonely and with enough contact increased by 39 % (97-135) p.32.<br><br>The **number of participants reported that** they have poor well-being (ratings of 1 or 2) has decreased from 92 to 51, and the number of those considering they have good well-being (ratings of 4 or 5) has increased from 46 to 108. | increase of 63 additional consultations in total, 34 had a decrease of 2,125 in total, and 1 had no change. Most health care providers reported the key benefit of SP to be reduction in participants' isolation and loneliness.<br>***A&E*:** 14% reduction in participants' attendance after 6 months,17% reduction after 12 months.<br>***Inpatient Admissions***<br>14% reduction after 6 months, 15% reduction after 12 months. | |
| **Programme name:** Social Cure[44] Same protocol as[39], slight difference in research focus.<br><br>**Location:** East Midlands UK. A relatively affluent suburban borough of East Midlands.<br><br>**Evaluated by:** Kellezi and colleagues, | **Loneliness:** 8-item UCLA Loneliness Scale (ULS-8).<br><br>**Social isolation:** Not assessed.<br><br>**Well-being:** Not assessed.<br><br>**Connectedness:** Community belonging: ('*Thinking about this local community, the kind of place it is and the kind of people who live around here,* | **Interviews** revealed that being a part of a group (family, community, volunteering group) and feeling that one belongs to a community helps people feel less lonely. Participants report that having a positive relationship with link workers has helped them build self-confidence, which in turn has helped them address their experiences of loneliness.<br><br>Participants' number of group memberships increased significantly between T0 (M =1.89, | Patients used primary care services less at T1 (n=797) than T0 (n=1063), with a 25% (n=266) reduction in appointments.<br><br>Participants' primary care use decreased significantly between T0 (*M*=5.9, *SD*=8.2) and T1 (*M*=4.5, *SD*=8.4), | **Change in** number of group memberships was a positive predictor of community belonging at a follow-up (T1, *Coeff*=0.09, *SE*=0.04, *t*=2.61, p=0.01, *LLCI*=0.02, *ULCI*=0.16). Increase in community belonging could be considered as an |

| | | | | |
|---|---|---|---|---|
| Nottingham Trent University | *would you say that you feel a sense of belonging to this local community*?'). <br><br> **Other measures:** <br> Number of group memberships: a list of 10 social groups to select from and a choice of 'not a member of any'. <br><br> Mixed method approach. Study 1: semi-structured interviews; Study 2: longitudinal survey. Pre/post programme comparisons. | SD =1.59) and T1 (M =2.21, SD =1.87, F (1,177)=5.34, p=0.022, partial η 2=0.029). **Change in** number of group memberships was a positive predictor of community belonging at a follow-up (T1, *Coeff*=0.09, *SE*=0.04, *t*=2.61, p=0.01, *LLCI*=0.02, *ULCI*=0.16), while community belonging at the follow-up was a negative predictor of loneliness at follow-up (T1 *Coeff*=−0.31, *SE*=0.07, *t*=−4.15, p=0.0001, *LLCI*=−0.45, *ULCI*=−0.16), which was a positive predictor of primary healthcare usage at follow-up(T1, *Coeff*=1.41, *SE*=0.45, *t*=3.13, p=0.002, *LLCI*=0.52, *ULCI*=2.31.). These findings indicate that individuals that are members of more groups are likely to have a greater sense of community belonging, lower loneliness (no loneliness) scores and are likely to use health care services less. | *F*(1,176)=9.14, p=0.003, with T1 (n=797) compared to T0 (n=1063). Overall 25% (n=266) reduction in primary care appointments. <br><br> GPs, health coaches, and link workers recognise the limitations of the 'traditional medical model', and express concerns over addressing loneliness with medical provisions. GPs view social prescribing as best model to address loneliness and reduce its negative health impacts. | aspect that could be used to assess the impact of social prescribing on a community-level. As the authors suggest, future studies in areas with fewer community resources/lower community cohesion could help with capturing this level of impact more clearly. Primary focus is on understanding how community resources can be used to reduce loneliness and improve social connectedness, to positively impact health care usage, and less so on impact of the programme on community. |
| **Programme name:** Connecting Communities[45] <br><br> **Location:** <br> 30 locations across the UK | **Loneliness:** 3-item UCLA loneliness scale. <br><br> **Social isolation:** Not assessed. <br><br> **Well-being:** short WEMWBS. <br><br> **Connectedness:** Not assessed. | **82 % of participants experiencing loneliness before programme start, resulting in 69% less lonely**, 27% no change; 4% more lonely at the end of the programme. Participants under 60 years old had more improvement in loneliness compared to those over 60. Greater impact on participants identified as being in a life | Not assessed. | A social return of £2.04 per £1 invested (based on running costs with set up costs removed). |

| | | | | |
|---|---|---|---|---|
| **Evaluated by:** Co-op and British Red Cross in collaboration with School of Health and Related Research at the University of Sheffield. | Surveys. Pre/post programme comparisons. | transition (health issues, mobility limitations, new child, recent bereavement, divorce/ separation, retirement, children moving out) than on those not experiencing transition.<br><br>**76% of participants reported improvement in well-being scores.** Participants reported improvements in self-esteem and confidence – linking it to improvements in well-being. | | |
| **Programme name:** No name[46]<br><br>**Location:** SOAR, Sheffield-based social prescribing organisation, UK<br><br>**Evaluated by:** Payne and colleagues University of Sheffield, UK. | **Loneliness:** Not assessed.<br><br>**Social isolation:** Not assessed.<br><br>**Well-being:** Not assessed.<br><br>**Connectedness:** No definition of the term or measure specified.<br><br>Semi-structured interviews involving people attending a range of social prescribing activities. Analysis used a thematic approach, in which emerging themes were contextualised with interview transcripts and findings from existing literature. | Participants report that the most significant benefits of being a part of the social prescribing in relation to connectedness were: increased interaction with individuals outside of their circle, improvements in and learning of new ways to relating to other people, enjoyment that comes from engaging with others, forging new connections. | Not assessed. | Not assessed. |
| **Programme name:** No name[47]<br><br>**Location:** Perth, Western Australia | **Loneliness:** Not assessed.<br><br>**Social isolation:** Not assessed.<br><br>**Well-being**: measure not specified.<br><br>**Connectedness:** Not assessed. | The report concludes that the physical exercise had a positive impact on well-being. Some of the benefits mentioned include participants reporting being more positive, with the new outlook on life extending to family members of the participants. | Not assessed. | Not assessed. |

| | | | | |
|---|---|---|---|---|
| **Evaluated by:** Raynor and colleagues at Edith Cowan University, Western Australia, and University of South Australia. | Interviews, pre and post comparisons, control group. | | | |
| **Programme name:** Live Lively[48]<br><br>**Location:** Veor Camborne Cornwall, UK<br><br>**Evaluated by:** Care@Veor | **Loneliness:** Not assessed.<br><br>**Social Isolation**: measure not specified.<br><br>**Well-being**: measure not specified.<br><br>**Connectedness:** Not assessed.<br><br>**Method not clear.** | Insufficient evidence to determine impact on social isolation and well-being. | Not assessed. | Not assessed. |
| **Programme name:** No name.[49]<br><br>**Location:** Plymouth and Manchester, UK<br><br>**Evaluated by:** Callaghan and colleagues at University of Plymouth, University of | **Loneliness:** Not assessed.<br><br>**Social isolation:** Not assessed.<br><br>**Well-being:** WEMWBS.<br><br>**Connectedness:** Not assessed.<br><br>Mixed-methods process evaluation: two-group randomised controlled trial; Semi -structured one-to-one interviews. Pre and post comparisons. | The mean between-group difference (intervention minus control) in the WEMWBS score was 4.6 (– 1.7 to 10.8) at 3 months, and 1.9 (95% CI – 4.6 to 8.4) at 6 months. P values are not reported as the aim of this particular trial report was to report the values for the main outcome variable, WEMWBS, at the 3- and 6-month follow-ups, which showed indicated some difference in favor of the intervention programme. These estimates are then to be | Not assessed. | Not assessed. |

| | | | | |
|---|---|---|---|---|
| Manchester, University of Exeter, Plymouth City Council, University of Southampton. | | used to provide estimates for a sample size calculation for a definitive trial. | | |
| **Programme name:** No name.[50]<br><br>**Location:** Large City in England, potentially, Bristol, UK<br><br>**Evaluated by:** Malyn at the University of the West of England, Bristol for the degree of Doctor of Counselling Psychology. | **Loneliness:** Not assessed.<br><br>**Social Isolation:** Issues explored in relation to 4 themes: 'Relationship to self', 'Relationship with others', 'Relationship to facilitator' and 'An intermediary object.<br><br>**Well-being:** Not assessed.<br><br>**Connectedness:** Not assessed.<br><br>Semi-structured interviews, thematic analysis. | Interview evidence suggests that reading and writing groups are effective as an intervention for reducing social isolation and loneliness in older adulthood. Gathering a rich account of the participants' experience of groups, rather than attempt to quantify the impact of participation. | Not assessed. | Not assessed. |
| **Programme name:** No name.[51]<br><br>**Location:** two regions of Norway | **Loneliness:** Not assessed.<br><br>**Social isolation:** Not assessed.<br><br>**Well-being:** defined in the terms of vitality and joy. | **When asked: 'how has being in a choir affected you?', all 19 participants found it significant for their well-being**. Eight of them referred to singing as vital to their well-being or even their survival.<br><br>Participants reported psychological effects: mood elevation, relief from anxiety, greater | Not assessed. | Not assessed. |

| | | | | |
|---|---|---|---|---|
| **Evaluated by:** Batt-Rawden and Andersen at the Norwegian University of Science and Technology (NTNU), Norway and Herstmonceux Integrative Health Centre, Hailsham, East Sussex, UK. | **Connectedness:** defined in the terms of belonging / identity and social inclusion / cohesion.<br><br>Choral singing can affect women's perceptions of their well-being in four distinct ways:<br>(i) through the joy of singing,<br>**(ii)** experiencing singing as essential for survival,<br>**(iii)** (iii) group singing as a route to **social connection**, which enhanced a sense of identity and of belonging, and thereby increased self-confidence and self-esteem and<br>**(iv)** through promoting social inclusion.<br><br>Focus groups, semi-structured interviews. ethno-graphic study. | emotional and self-awareness, and, **mostly, they talked about 'connection'. S**inging provided them with social connection, giving them **a sense of belonging in the choir**, and enhancing the way that they related to family and colleagues. Singing as a bridgebuilding activity constructing social connectedness and belonging in a community. | | |
| **Programme name:** No name.[52]<br><br>**Location:** London, UK.<br><br>**Evaluated by:** Thomson, and colleagues at UCL. | **Loneliness:** Not assessed.<br><br>**Social Isolation:** interview quotes, observed in the terms of: connections with group members; routine and structure of getting out of the house.<br><br>**Well-being:** UCL Museum well-being Measure, a positive mood scale where | **Participants felt that the intervention gave them routine and structure** with an opportunity to engage positively with others, which in turn **decreased the sense of social isolation.**<br>Pre–post comparisons show a stat.sig. increase in **well-being scores,** $t(19)=6.96$, $p<.001$. | Not assessed. | The formation of communities of practice. |

| | | | | |
|---|---|---|---|---|
| | participants rate each of six mood items (Active, Alert, enthusiastic, excited, Happy and inspired) on a 5-point scale<br>1 = 'i don't feel';<br>2 = 'i feel a little bit';<br>3 = 'i feel fairly';<br>4 = 'i feel quite a bit'; and<br>5 = 'i feel extremely<br><br>**Connectedness:** interview quotes, defined as a building a sense of community: group formation, connections, shared experiences, learning, positive mood.<br><br>Semi-structured interviews and diaries, surveys. Pre and post programme comparisons. | Across interviews, participants described how the programme had fostered a **sense of community** over the 10 weeks. Participants noted how the sense of community was facilitated by a number of related characteristics of the programme, first **from knowledge and reassurance of taking part in activities with other people with shared experience of mental health difficulties**. Second, the programmes **provided new, hands-on skills** in both horticulture and arts-based practice, and this learning appeared to contribute to building a sense of community. | | |
| **Programme name:** Evaluation of the Hale, Community Connectors Social Prescribing service[53]<br><br>**Location:** Hale, UK<br><br>**Evaluated by:** Dayson and Leather, Centre for Regional Economic and Social Research Sheffield Hallam University | **Loneliness:** Not assessed.<br><br>**Social isolation:** Not assessed.<br><br>**Well-being:** SWEMWBS.<br><br>**Connectedness:** friendships, engagement with others and reliance.<br><br>Case studies, surveys, pre and post programme comparisons. | Well-being of almost **three-quarters of service users** improved following their referral, with average **SWEMWBS score** from 18 to 22.<br><br>Connectedness and social relationships of many service users improved after the participation. There were increases in the proportion of service users content with their friendships and relationships, saying they have enough people the feel comfortable asking for help at any time, and saying their relationships are as satisfying as they would want them to be. | 7% fewer GP attendances.<br>9% fewer A&E visits.<br><br>The data on health usage is inconsistent in relation to how events are recorded. | Not assessed. |

| | | | | |
|---|---|---|---|---|
| **Programme name:** Bolton CVS Community Asset Navigator Programme[54]<br><br>**Location:** Bolton<br><br>**Evaluated by:** Bolton CVS. | **Loneliness:** Not assessed.<br><br>**Social isolation:** Not assessed.<br><br>**Well-being:** measure not specified.<br><br>**Connectedness:** measure not specified.<br><br>Case studies and surveys. Pre/post. | 95% of participants felt more connected. Levels increased from 1.47 to 3.21.<br><br>71% of participants saw an increase in their mental health and well-being. Levels increased from 2.01 to 3.05. | Not assessed. | Not assessed. |
| **Programme name:** Age UK's Cascade Training Programme Evaluation Report[55]<br><br>**Location:** Across England, UK<br><br>**Evaluated by:** Alden and colleagues, University of Leeds | **Loneliness:** measure not specified.<br><br>**Social isolation:** Not assessed.<br><br>**Well-being:** measure not specified.<br><br>**Connectedness:** Not assessed.<br><br>Interviews, surveys, focus groups, documentary analysis, follow-up with organisations' data collection teams. | Service delivery staff report positive impact of social prescribing on loneliness, recommended that training manuals include measures to address loneliness and social isolation. 95% of staff report ability to support more older people as a direct result of the program. 58% of volunteers report positive impact on their own mental health and well-being. | Positive impact on care home services, improving residents' quality of life. | Delivery organisations report expanding services and creating new activities due to program. Programme brought together housing associations, sheltered housing and care home staff, health care providers, faith-based organisations, and local charities, which has a positive impact on community engagement. Participants report interest in helping others and sharing information, thereby expanding community |

| | | | | |
|---|---|---|---|---|
| | | | | capacity to respond to challenges. |
| **Programme name:** Culture Vitamins – an Arts on Prescription Project in Denmark[56]<br><br>**Location:** Aldborg, Denmark<br><br>**Evaluated by:** Jensen, Aalborg University | **Loneliness:** Not assessed<br><br>**Social Isolation:** Not assessed<br><br>**Well-being:** joy, vitality, motivation.<br><br>**Connectedness:** Not assessed<br><br>Qualitative, semi-structured interviews.<br><br>Open questions elicited participants' experiences of attending the AoP project in Aldborg.<br><br>A thematic approach was applied to analyse the data and a theoretical lens of Salutogenesis was employed to explore the findings.<br><br>The themes that emerged from the data describe the participants' subjective experiences and were (1) positive changes, (2) overcoming challenges and being in the 'space' and (3) moving from self-critical to self-caring. | The majority of the participants reported that their mental health improved after participating in the project. The reported mental health benefits were increased energy level, increased self-esteem, more joy in life, less panic attacks, increased motivation, a better understanding of own needs, an increased level of self-care.<br><br>One participant described how he has become more interested in joining the world and interacting with other people.<br><br>One participant commented that it was a big leap for her to start 'Culture Vitamins' after a long period of being indoors and not engaging in new social relationships, but it gradually improved.<br><br>The participants have experienced a positive change in their well-being, including improved energy, decrease in anxiety attacks and feeling that trying to manage a job is a real possibility. | Not assessed. | Some of the participants said that they felt more aligned to the labour market. |

**References**


1. Bird EL, Biddle MSY, Powell JE. General practice referral of 'at risk'populations to community leisure services: applying the RE-AIM framework to evaluate the impact of a community-based physical activity programme for inactive adults with long-term conditions. BMC Public Health. 2019;19(1):1308.

2. Wigfield A, Kispeter E, Alden S, Turner R, Clarke T. Age uk's fit for the future project: Evaluation report. Circ Leeds Available Www Ageuk Org Ukhealth-Wellbeingfit---Fiddlefit---Futur 27 Sept 2016 [Internet]. 2015; Available from: https://www.sheffield.ac.uk/polopoly_fs/1.569567!/file/fit-for-the-future-Final-Evaluation.pdf

3. Hughes ME, Waite LJ, Hawkley LC, Cacioppo JT. A Short Scale for Measuring Loneliness in Large Surveys. Res Aging. 2004;26(6):655–72.

4. Grant C, Goodenough T, Harvey I, Hine C. A randomised controlled trial and economic evaluation of a referrals facilitator between primary care and the voluntary sector. Bmj. 2000;320(7232):419–23.

5. Moffatt S, Steer M, Lawson S, Penn L, O'Brien N. Link Worker social prescribing to improve health and well-being for people with long-term conditions: qualitative study of service user perceptions. BMJ Open. 2017 Jul 1;7(7):e015203.

6. Wildman JM, Moffatt S, Steer M, Laing K, Penn L, O'Brien N. Service-users' perspectives of link worker social prescribing: a qualitative follow-up study. BMC Public Health. 2019;19(1):98.

7. Mercer SW, Fitzpatrick B, Grant L, Chng NR, McConnachie A, Bakhshi A, et al. Effectiveness of Community-Links Practitioners in Areas of High Socioeconomic Deprivation. Ann Fam Med. 2019 Nov;17(6):518–25.

8. Hanlon P, Gray CM, Chng NR, Mercer SW. Does Self-Determination Theory help explain the impact of social prescribing? A qualitative analysis of patients' experiences of the Glasgow 'Deep-End' Community Links Worker Intervention. Chronic Illn. 2019 May 3;1742395319845427.

9. Sheffield Hallam University and Sport Industry Research Centre. Dance to Health "Phase 1 rollout [test and learn]" evaluation [Internet]. 2019. Available from: https://ae-sop.org/wp-content/uploads/sites/63/2019/06/SHU-SIRC-1st-report-FINAL.pdf

10. Carnes D, Sohanpal R, Frostick C, Hull S, Mathur R, Netuveli G, et al. The impact of a social prescribing service on patients in primary care: a mixed methods evaluation. BMC Health Serv Res. 2017 Dec 19;17(1):835.

11. Paterson C. Measuring outcomes in primary care: a patient generated measure, MYMOP, compared with the SF-36 health survey. BMJ. 1996 Apr 20;312(7037):1016–20.

12. Poulos RG, Marwood S, Harkin D, Opher S, Clift S, Cole AMD, et al. Arts on prescription for community-dwelling older people with a range of health and wellness needs. Health Soc Care Community. 2018;27(2):483–92.

13. Bell A, Gandy RJ, Roe B. Benefits and impacts of Active Lives groups for older people living in the community. Res Policy Plan J Soc Serv Res Group. 2017;32(2):97–112.

14. Bowling A. Ageing well: Quality of life in old age. McGraw-hill education (UK); 2005.

15. Elston J, Gradinger F, Asthana S, Lilley-Woolnough C, Wroe S, Harman H, et al. Does a social prescribing 'holistic' link-worker for older people with complex, multimorbidity improve well-being and frailty and reduce health and social care use and costs? A 12-month before-and-after evaluation. Prim Health Care Res Dev [Internet]. 2019 Sep 24 [cited 2020 Oct 29];20. Available from: https://www.ncbi.nlm.nih.gov/pmc/articles/PMC6764188/

16. Dayson C, Bennett E. Evaluation of the Rotherham Mental Health Social Prescribing Service 2015/16-2016/17 [Internet]. 2017. Available from: https://www4.shu.ac.uk/research/cresr/sites/shu.ac.uk/files/eval-rotherham-mental-health-social-prescribing.pdf

17. Dayson C, Damm C. The Rotherham Social Prescribing Service for People with Long-term Conditions: Evaluation Update [Internet]. 2017. Available from: https://www4.shu.ac.uk/research/cresr/sites/shu.ac.uk/files/rotherham-social-prescribing-evaluation-update.pdf

18. Dayson C, Damm C. Evaluation of the Rotherham Social Prescribing Service for Long Term Conditions. 2020;18.

19. Woodall J, Trigwell J, Bunyan A-M, Raine G, Eaton V, Davis J, et al. Understanding the effectiveness and mechanisms of a social prescribing service: a mixed method analysis. BMC Health Serv Res. 2018;18(1):N.PAG-N.PAG.

20. Kimberlee R. Gloucestershire Clinical Commissioning Group Social Prescribing Service: Evaluation Report [Internet]. 2016. Available from: https://future.nhs.uk/connect.ti/socialprescribing/view?objectId=40784069

21. Wessex Academic Health Science Network (WAHSN) and R-Outcomes. Social Prescribing in Wessex Understanding its impact and supporting spread [Internet]. 2017. Available from: https://wessexahsn.org.uk/img/projects/Wessex%20Social%20Prescribing-1529938576.pdf

22. Farenden C, Mitch C, Feast S, Verdenicci S. Community Navigation in Brighton & Hove Evaluation of a social prescribing pilot. 2015;68.

23. Vogelpoel N, Jarrold K. Social prescription and the role of participatory arts programmes for older people with sensory impairments. J Integr Care. 2014;22(2):39–50.

24. Stickley T, Hui A. Social prescribing through arts on prescription in a U.K. city: participants' perspectives (part 1). Public Health. 2012;126(7):574–9.

25. Whitelaw S, Thirlwall C, Morrison A, Osborne J, Tattum L, Walker S. Developing and implementing a social prescribing initiative in primary care: insights into the possibility of normalisation and sustainability from a UK case study. Prim Health Care Res Dev. 2017;18(2):112–21.

26. Pescheny J, Pappas Y, Randhawa G. Service user outcomes of a social prescribing programme in general practice. Int J Integr Care. 2018;18(s2):215.

27. Holding E, Thompson J, Foster A, Haywood A. Connecting communities: A qualitative investigation of the challenges in delivering a national social prescribing service to reduce loneliness. Health Soc Care Community. 2020;28(5):1535–43.

28. Jacob C, Roff K, Fleet J, Jones J, Wood CA, Jensen H, et al. CAN VOLUNTEERS IMPROVE THEWELL-BEING, PARTICIPATION AND ACTIVITY OF PATIENTS ON AN ACUTE OLDER-PERSONS' UNIT IN HOSPITAL?...British Geriatrics Society Autumn Meeting, November 6-8, 2019, Leicester, England. Age Ageing. 2020 Feb 2;49:i14–i14.

29. Aggar C, Thomas T, Gordon C, Bloomfield J, Baker J. Social Prescribing for Individuals Living with Mental Illness in an Australian Community Setting: A Pilot Study. Community Ment Health J [Internet]. 2020 May 13; Available from: http://search.ebscohost.com/login.aspx?direct=true&db=mnh&AN=32399601&site=ehost-live

30. Leslie C, McGill G, Kiernan MD, Wilson G. Social isolation and loneliness of UK veterans: a Delphi study. Occup Med Oxf Engl. 2020 Sep 9;70(6):407–14.

31. Envoy Partnership. Self-Care Social Prescribing [Internet]. 2018. Available from: https://www.kcsc.org.uk/sites/kcsc.org.uk/civi_files/files/civicrm/persist/contribute/files/Self%20Care/7641_SROI-Report_DIGITAL_AW.pdf

32. DAYSON, C. and BASHIR, N. The social and economic impact of the Rotherham Social Prescribing Pilot: Main evaluation report. [Internet]. Sheffield, Sheffield Hallam University. 2014 [cited 2020 Feb 13]. Available from: https://www4.shu.ac.uk/research/cresr/sites/shu.ac.uk/files/social-economic-impact-rotherham.pdf

33. Dayson C. Evaluating social innovations and their contribution to social value: the benefits of a "blended value" approach [Internet]. 2017 [cited 2020 Feb 12]. Available from: https://www.ingentaconnect.com/content/tpp/pap/2017/00000045/00000003/art00006;jsessionid=5ffokecerfpb6.x-ic-live-03

34. Dayson C, Bennett E. Evaluation of Doncaster Social Prescribing Service: understanding outcomes and impact. 2016;34.

35. Kimberlee R., Ward R., Jones M., and Powell J. Measuring the economic impact of Wellspring Healthy Living Centre's Social Prescribing Wellbeing Programme for low level mental health issues encountered by GP services. 2014;111.

36. Holt NJ. Tracking momentary experience in the evaluation of arts-on-prescription services: using mood changes during art workshops to predict global wellbeing change. Perspect Public Health. 2020 Sep 1;140(5):270–6.

37. Wilkinson EK, Lees A, Weekes S, Duncan G, Meads G, Tapson K. A collaborative, multi-sectoral approach to implementing a social prescribing initiative to alleviate social isolation and enhance well-being amongst older people. J Integr Care [Internet]. 2020 [cited 2020 Nov 16];ahead-of-print(ahead-of-print). Available from: https://doi.org/10.1108/JICA-02-2020-0004

38. Heijnders ML, Meijs JJ. 'Welzijn op Recept' (Social Prescribing): a helping hand in re-establishing social contacts – an explorative qualitative study. Prim Health Care Res Dev. 2018 May;19(3):223–31.

39. Wakefield JRH, Kellezi B, Stevenson C, McNamara N, Bowe M, Wilson I, et al. Social Prescribing as 'Social Cure': A longitudinal study of the health benefits of social connectedness within a Social Prescribing pathway. J Health Psychol. 2020 Jul 23;1359105320944991.

40. Todd C, Camic PM, Lockyer B, Thomson LJM, Chatterjee HJ. Museum-based programs for socially isolated older adults: Understanding what works. Health Place. 2017 Nov;48:47–55.

41. Bellis A, Haylen A, Foster D, Grimwood GG, Hutton G, Woodhouse J, et al. Tackling loneliness. 2020 Oct 26 [cited 2020 Oct 26]; Available from: https://commonslibrary.parliament.uk/research-briefings/cbp-8514/

42. Redmond M, Sumner RC, Crone DM, Hughes S. 'Light in dark places': exploring qualitative data from a longitudinal study using creative arts as a form of social prescribing. Arts Health. 2018;11(3):232–45.

43. Waterfall D. Dudley CVS: Integrated Plus Impact Evaluation [Internet]. 2019 p. 107. Available from: https://future.nhs.uk/socialprescribing/view?objectId=48482725

44. Kellezi B, Wakefield JRH, Stevenson C, McNamara N, Mair E, Bowe M, et al. The social cure of social prescribing: a mixed-methods study on the benefits of social connectedness on quality and effectiveness of care provision. BMJ Open [Internet]. 2019 Nov 1 [cited 2020 Jan 26];9(11). Available from: https://bmjopen.bmj.com/content/9/11/e033137

45. Coop and British Red Cross. Tackling-loneliness-and-isolation-connecting-communities.pdf [Internet]. 2019. Available from: https://www.sheffield.ac.uk/media/6027/download

46. Payne K, Walton E, Burton C. Steps to benefit from social prescription: a qualitative interview study. Br J Gen Pract. 2020 Jan 1;70(690):e36–44.

47. Raynor AJ, Iredale F, Crowther R, White J, Dare J. It's Not Just Physical: Exercise Physiologist-Led Exercise Program Promotes Functional and Psychosocial Health Outcomes in Aged Care. J Aging Phys Act. 2020 01;28(1):104–13.

48. Primary Care@Veor. Live Lively'What matters to me', not 'what is the matter with me'. 2019.

49. Callaghan L, Thompson TP, Creanor S, Quinn C, Senior J, Green C, et al. Individual health trainers to support health and well-being for people under community supervision in the criminal justice system: the STRENGTHEN pilot RCT [Internet]. Southampton (UK): NIHR Journals Library; 2019 [cited 2020 Nov 29]. (Public Health Research). Available from: http://www.ncbi.nlm.nih.gov/books/NBK551448/

50. Malyn B. Reading and writing for well-being: A qualitative exploration of the therapeutic experience of older adult participants in a bibliotherapy and creative writing group. 2018 Jun 19 [cited 2020 Nov 29]; Available from: https://uwe-repository.worktribe.com/output/866482/reading-and-writing-for-well-being-a-qualitative-exploration-of-the-therapeutic-experience-of-older-adult-participants-in-a-bibliotherapy-and-creative-writing-group

51. Batt-Rawden K, Andersen S. 'Singing has empowered, enchanted and enthralled me'-choirs for wellbeing? Health Promot Int. 2020;35(1):140–50.

52. Thomson LJ, Morse N, Elsden E, Chatterjee HJ. Art, nature and mental health: assessing the biopsychosocial effects of a "creative green prescription" museum programme involving horticulture, artmaking and collections. Perspect Public Health. 2020 Sep;140(5):277–85.

53. Dayson C, Leather D. Evaluation of Hale Community Connectors Social Prescribing Service 2017 [Internet]. 2018. Available from: https://www4.shu.ac.uk/research/cresr/sites/shu.ac.uk/files/eval-HALE-community-connectors-social-prescribing.pdf

54. Bolton CVS. Community Asset Navigator Programme: Reach, Progress and Outcomes Report [Internet]. 2019. Available from: https://www.boltoncvs.org.uk/sites/boltoncvs.org.uk/files/CANs%20Q4%20Report%20-Print.pdf

55. Alden S, Wigfield A, Kispeter E. Age UK's Cascade Training Programme Evaluation Report. Circ Cent Int Res Care Labour Equal Univ Leeds. 2015;60.

56. Jensen A. Culture Vitamins – an Arts on Prescription project in Denmark: Perspect Public Health [Internet]. 2019 Apr 8 [cited 2020 Nov 30]; Available from: https://journals.sagepub.com/doi/10.1177/1757913919836145
